# LC-ESI(±)-LTQ MS^n^‑Based Metabolomic Profiling of Coffee: Fragmentation Pathways for Identification of Major Polar Compounds

**DOI:** 10.1021/jasms.4c00418

**Published:** 2025-05-13

**Authors:** Marcos Valério Vieira Lyrio, Danieli Grancieri Debona, Amanda Eiriz Feu, Nayara Araujo dos Santos, Arlan da Silva Gonçalves, Ricardo Machado Kuster, Eustáquio Vinícius Ribeiro de Castro, Wanderson Romão

**Affiliations:** † 28126 Federal University of Espírito Santo/UFES, Department of Chemistry, Campus Goiabeiras, Avenida Fernando Ferrari, 514, 29075-910 Vitoria, Espírito Santo, Brazil; ‡ Federal Institute of Espírito Santo − Campus Vila Velha, Av. Ministro Salgado Filho, Soteco, 29106-010 Vila Velha, Espírito Santo, Brazil

**Keywords:** Mass spectrometry, Liquid chromatography, Tandem MS, Linear ion trap, Coffee

## Abstract

Coffee is characterized by a complex chemical matrix that significantly influences its organoleptic properties and market value. This complexity is driven by factors such as botanical species, geographical origin, cultivation conditions, and post-harvest processing methods. Metabolomic studies aim to elucidate how these factors impact the biosynthesis of metabolites that contribute to the sensory qualities of high-quality coffee. Among various analytical techniques, liquid chromatography-mass spectrometry (LC-MS) is particularly effective for separating, identifying, and quantifying these compounds. Most metabolomic studies employ high-resolution mass spectrometry (HRMS) for its superior mass accuracy (<1 ppm), whereas the interpretation of low-resolution data requires additional effort, often relying on literature references and proposed fragmentation mechanisms. In this study, we applied LC-ESI(±)­LTQ MS^n^ to comprehensively profile coffee metabolites, identifying 60 compounds, including polar compounds and their isomers such as chlorogenic acids, carbohydrates, amino acids, alkaloids, glycosylated diterpenes, and flavonoids. Fragmentation mechanisms were proposed and discussed. The results demonstrate the effectiveness of LC-ESI(±)­LTQ MS^n^ in a detailed metabolomic analysis, providing a robust platform for future research in coffee metabolomics.

## Graphical abstract

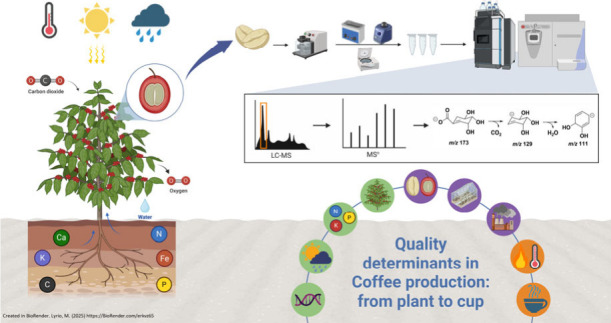

## 1 Introduction

The chemical complexity of coffee significantly influences its sensory attributes, which are the major determinants of its market value. This complexity arises from multiple factors, including botanical species, geographical origin, cultivation conditions, and post-harvest processes.^1^ Understanding how these factors affect the chemical profile of coffee is essential for developing methods and processes to enhance the quality and consistency of the product.^2^

Therefore, green coffee has emerged as a focal point for metabolomic studies, particularly as a subject of analysis using liquid chromatography-mass spectrometry (LC-MS). This technique enables the exploration of correlations between chemical composition and sensory attributes, as well as the impact of several processing methods and biosynthesis of metabolites that contribute to the sensory attributes of high-quality coffees.^3,4^

Several studies have successfully identified and quantified diverse classes of compounds in coffee through high-resolution mass spectrometry (HRMS) and low-resolution mass spectrometry (LRMS) platforms.^5–7^ These techniques have been explored in Omics Sciences, particularly metabolomics, which involves the comprehensive study of metabolites in a biological system. This includes the identification and quantification of compounds that are either end products or intermediates of cellular metabolism. In this context, metabolomics aims to understand metabolic responses to physiological, environmental, or pathological conditions which is suitable to understand the factors that determine coffee quality.^8–10^

Mass spectrometry can identify metabolites through two main approaches. The first, using HRMS, begins by obtaining exact mass measurements and isotopic patterns to screen possible molecular formulas, which facilitates the identification procedure. The ion of interest is then fragmented using MS/MS or MS^n^, and the fragmentation patterns are compared with spectral/compound libraries and the predicted molecular formula.^11,12^ The second approach, used in LRMS, does not allow exact mass measurements and focuses directly on fragmentation experiments. The identification is based on matching reference spectra, consulting literature specific to or similar to the studied matrix, and proposing fragmentation mechanisms. Although LRMS offers lower mass accuracy, it is effective and affordable, particularly for laboratories with limited resources or for routine analyses.^6,13^

In this context, HRMS has been extensively explored for green coffee analysis, in both metabolomic and lipidomic studies, especially using orbitrap and time-of-flight (TOF) analyzers. These studies include correlations between chemical profiles and factors such as maturation levels, post-harvest processing methods, botanical origin, and determination of quality-related markers.^14–17^

In contrast, several applications have emerged by using LRMS, particularly for targeted studies. Aurum et al. (2022)^18^ identified 84 lipids in *Coffea arabica* and *Coffea canephora* from Indonesia. Jaiswal et al. (2010)^6^ conducted extensive research on the profiling of chlorogenic acids using ion trap mass analyzers and sequential fragmentation experiments (MS^n^), leading to the identification of over 70 chlorogenic acids and their derivatives in *Coffea canephora* var. robusta. However, this study was limited to a single chemical class and relied on a more labor-intensive Soxhlet extraction method, which required larger sample volumes (5 g), greater amounts of solvent, and longer extraction times (5 h). Although the study was based on an ion trap analyzer, it was supported by a TOF mass spectrometer.

Clifford et al. (2003)^5^ described the fragmentation behavior of 18 chlorogenic acids using ion trap mass analyzers, providing reference fragmentation spectra for these compounds (MS^n^). In subsequent studies, Clifford et al. (2006)^13,19^ described the fragmentation behavior of dicaffeoylquinic acids and other hydroxycinnamic acid derivatives, where 45 compounds were identified in *Coffea canephora* var. robusta. Cinnamoyl–amino acid conjugates have also been identified in robusta, using ion trap analyzers, with seven compounds reported in total.^20^ These studies confirm the capability of ion trap analyzers for the identification of compounds in coffee. However, they also highlight the need for further investigation focusing on low-resolution mass spectrometry and new fragmentation studies. Despite the progress made, there is still a gap in the literature regarding the fragmentation behavior of other classes of compounds and detailed descriptions of their fragmentation pathways.

Therefore, the present study utilizes LC-ESI(±)­LTQ MS^n^ based on the multistage fragmentation experiments to characterize the metabolomic profile of coffee and propose fragmentation mechanisms for all identified classes. This approach emphasizes qualitative analysis and fragmentation studies, proposing a straightforward and detailed procedure for the identification of major polar compounds and their isomers. The results are compared with existing literature on coffee and related matrixes, thereby contributing to a more comprehensive understanding of coffee’s chemical complexity and extending the applicability of LRMS in metabolomic research.

## 2 Materials and Methods

### 2.1 Extraction Procedure

Green coffee samples of *Coffea canephora* var. conilon and *Coffea arabica* from the State of Espírito Santo, Brazil, underwent natural processing and were subsequently separated and classified according to the Official Brazilian Classification.^21^

For metabolomic analysis, green coffee beans were ground in a knife mill to obtain a particle size of 20 mesh (0.85 mm). Subsequently, 100 mg of the ground coffee was transferred to 2 mL Eppendorf microtubes, to which 1.5 mL of a 50:50 mixture of methanol (LC-MS grade, LiChrosolv, Sigma-Aldrich, USA) and ultrapure water (Synergy Merck, Germany) were added. The microtubes were then subjected to ultrasonic bath treatment and vortex agitation for 30 min each. Following this, the microtubes were centrifuged for 10 min under 10,000 rpm. The resulting supernatant was filtered through a 0.22 μm syringe filter. Next, 20 μL of the crude extract was diluted in 980 μL of a methanol/water solution (50:50).

### 2.2 LC-ESI­(±)­LTQ MS

#### 2.2.1 Instrumental Conditions

The LC-MS analysis was performed using a UHPLC Vanquish system (Thermo Scientific, USA) equipped with a reversed-phase Agilent InfinityLab Poroshell 120 EC-C18 (2.1 × 150 mm^2^, 1.9 μm particle size). Chromatographic separation employed a biphasic mobile phase approach: mobile phase A consisted of water, and mobile phase B consisted of methanol, both containing 0.1% v/v formic acid (Sigma-Aldrich, USA). Solvent elution occurred at a flow rate of 0.25 mL/min following a gradient profile, starting with 20% B and increasing to 50% B over 27 min, followed by an increase to 90% B in 5 min. This condition was maintained for 3 min before returning to the initial condition (20% B), which was maintained for an additional 3 min, resulting in a total run time of 38 min. The column temperature was set at 40 °C with an injection volume of 5 μL.

For data acquisition, an LTQ XL mass spectrometer (Thermo Fisher Scientific, USA) was used with an electrospray ionization (ESI) source operating in positive and negative ionization modes. The mass acquisition range was set between *m*/*z* 50 and 1000. The ESI source parameters were set as follows: (i) source voltage = 3.5 kV (−5 kV in negative ionization mode); (ii) source temperature = 300 °C; (iii) sheath gas flow rate = 35 arbitrary units (a.u.); (iv) auxiliary gas flow rate = 10 au; (v) capillary voltage = 3 V (−33 V in negative ionization mode); and (vi) capillary inlet temperature = 350 °C.

#### 2.2.2 Data Analysis

The compounds were identified using top-3 data-dependent acquisition (DDA) ion tree experiments with MS^2^ and MS^3^ fragmentations. Precursor ion selection was performed with a 1 Da isolation window, and analyte fragmentation was achieved with a collision energy (CID) of 35%, consistent with other studies in the literature.^5,6,13^ Dynamic exclusion was enabled with a 30 s exclusion window. Fragmentation mechanisms were proposed, and isomers were distinguished based on fragment intensity, stability, and retention time. Additionally, spectral libraries, such as NIST, and references from the literature were consulted to support the compound identification. A total of 238 features were detected in ESI(−) and 130 in ESI­(+). The data were processed using the Xcalibur software version 4.2.47 (Thermo Fisher Scientific, USA).

##### 2.2.2.1 DFT Simulation of Proton Transfer in Chlorogenic Acids

Although this work is predominantly experimental, it was necessary to simulate the proton transfer in the first step of the proposed mechanism. This simulation was crucial since the proton transfer for this molecule has pathways which involve the transfer from a phenolic hydrogen to a carbonyl group—both of which are relatively distant from each other in the molecule.

The 3D structure of the reactant (chlorogenic acid) was obtained from the PubChem database. Subsequently, a random conformational analysis was performed using the GHEMICAL software,^22^ employing the TRIPOS 5.2 force field.^23^ The conformer with the lowest energy was then optimized using ORCA 5.04 ^24^ with the B3LYP/6-31G** DFT method.^25,26^

In parallel, the possible product molecule was edited by removing the proton from the hydroxyl group at the “*para*” position, and the single bond was replaced with a double bond. The same proton was then added to the oxygen atom of the carbonyl group to create a potential reaction product. This product followed the same optimization steps as for the reagent molecule.

Subsequently, a saddle point search was conducted by utilizing the coordinates of the reagents and products. The saddle point was then optimized by using the ORCA with the OPT-TS keyword. Finally, Hessian matrix calculations for the reagent, product, and transition state were performed with the NumFreq keyword to determine the Gibbs free energy and to obtain the vibrational frequencies in the infrared region.

## 3 Results and Discussion

The analysis of crude coffee extracts resulted in the typical chromatogram presented in Figure 1. A total of 60 compounds were identified, including classes such as chlorogenic acids, carbohydrates, amino acids, alkaloids, glycosylated diterpenes, and flavonoids in positive and negative ionization modes. The compounds were identified based on the analysis of MS^2^ and MS^3^ fragmentation spectra (Table 1).

**Figure 1:**
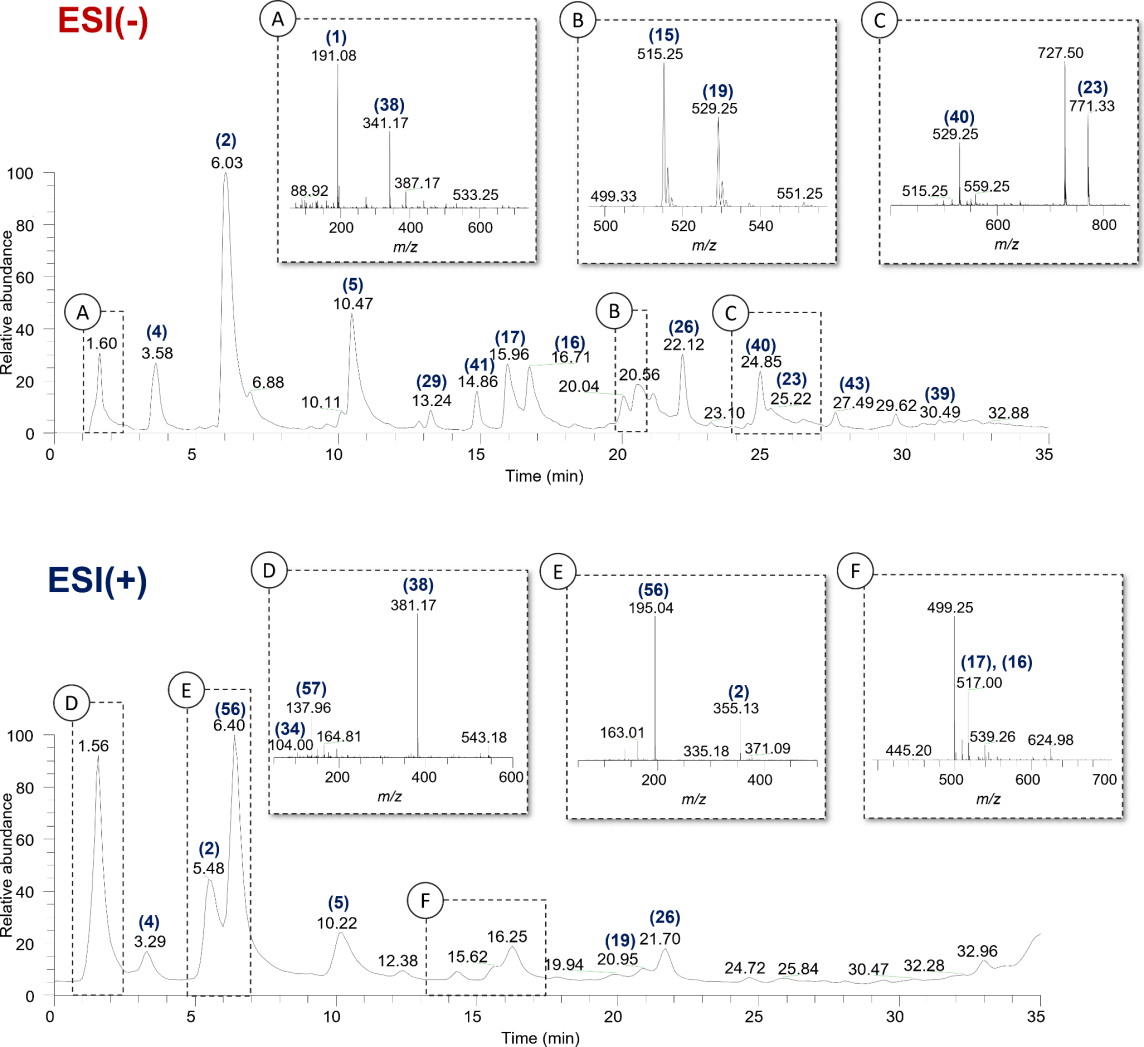
Typical chromatograms of coffee extracts, with selection (dashed line) of the identified peaks with their respective mass spectra in negative ionization mode (ESI(−)): **A** - (1) Quinic acid, (38) Sucrose; **B** - (15) 4,5-di*-O-*caffeoylquinic acid, (19) 3*-O-*caffeoyl-4*-O-*feruloylquinic acid; and **C** - (40) Carboxyatractyloside I, (23) 4*-O-*caffeoyl-5*-O-*feruloylquinic acid; and in positive ionization mode (ESI­(+)): **D** - (34) γ-Aminobutyric acid, (38) Sucrose, (57) Trigonelline; **E** - (56) Caffeine; and **F** - (16) 3,5-di*-O-*caffeoylquinic acid, (17) 3,4-di*-O-*caffeoylquinic acid.

**Table 1:** List of Compounds Identified by LC-ESI(±)­LTQ MS^n^

**ID**	***t***_**R**_ **(min)**	**Compound**	**Abbreviation** ^a^	Ion	***m*/*z***
1	1.60	Quinic acid	n.a.	[M – H]^−^	191
				[M + H]^+^	193
2	5.97	5*-O-*Caffeoylquinic acid	5-CQA	[M – H]^−^	353
				[M + H]^+^	355
3	6.91	4*-O-*Caffeoylquinic acid	4-CQA	[M – H]^−^	353
				[M + H]^+^	355
4	3.58	3*-O-*Caffeoylquinic acid	3-CQA	[M – H]^−^	353
				[M + H]^+^	355
5	10.47	5*-O-*Feruloylquinic acid	5-FQA	[M – H]^−^	367
				[M + H]^+^	369
6	10.57	4*-O-*Feruloylquinic acid	4-FQA	[M – H]^−^	367
				[M + H]^+^	369
7	5.92	3*-O-*Feruloylquinic acid	3-FQA	[M – H]^−^	367
				[M + H]^+^	369
8	9.60	5*-O-p*-Coumaroylquinic acid	5-pCQA	[M – H]^−^	337
9	9.84	4*-O-p*-Coumaroylquinic acid	4-pCQA	[M – H]^−^	337
10	5.07	3*-O-p*-Coumaroylquinic acid	3-pCQA	[M – H]^−^	337
11	25.06	3-*O*-Dimethoxycinnamoyl-5-*O*-caffeoylquinic acid	3D-5CQA	[M – H]^−^	543
12	32.06	4-*O*-Dimethoxycinnamoyl-5-*O*-feruloylquinic acid	4D-5FQA	[M – H]^−^	557
13	24.76	4-*O*-Sinapoyl-3-*O*-caffeoylquinic acid	4Si-3CQA	[M – H]^−^	559
14	21.02	3-*O*-Sinapoyl-5-*O*-caffeoylquinic acid	3Si-5CQA	[M – H]^−^	559
15	20.50	4,5-Di*-O-*caffeoylquinic acid	4,5-diCQA	[M – H]^−^	515
				[M + H]^+^	517
16	16.71	3,5-Di*-O-*caffeoylquinic acid	3,5-diCQA	[M – H]^−^	515
				[M + H]^+^	517
17	15.96	3,4-Di*-O-*caffeoylquinic acid	3,4-diCQA	[M – H]^−^	515
				[M + H]^+^	517
18	29.62	4,5-Di*-O-*feruloylquinic acid	4,5-diFQA	[M – H]^−^	543
19	20.01	3*-O-*Caffeoyl-4*-O-*feruloylquinic acid	3C-4FQA	[M – H]^−^	529
				[M + H]^+^	531
20	21.12	3*-O-*Caffeoyl-5*-O-*feruloylquinic acid	3C-5FQA	[M – H]^−^	529
				[M + H]^+^	531
21	24.69	4*-O-*Feruloyl-5*-O-*caffeoylquinic acid	4F-5CQA	[M – H]^−^	529
				[M + H]^+^	531
22	20.10	3*-O-*Feruloyl-5*-O-*caffeoylquinic acid	3F-5CQA	[M – H]^−^	529
				[M + H]^+^	531
23	25.25	4*-O-*Caffeoyl-5*-O-*feruloylquinic acid	4C-4FQA	[M – H]^−^	529
				[M + H]^+^	531
24	19.68	3*-O-p*-Coumaroyl-4*-O-*caffeoylquinic acid	3-*p*Co-4CQA	[M – H]^−^	499
25	24.83	*p*-Coumaroyl tryptophan	n.a.	[M – H]^−^	349
				[M + H]^+^	351
26	22.32	Caffeoyl tryptophan	n.a.	[M – H]^−^	365
				[M + H]^+^	367
27	26.39	Feruloyl tryptophan	n.a.	[M – H]-	379
				[M + H]^+^	381
28	16.21	*p*-Coumaroyl tyrosine	n.a.	[M – H]^−^	326
				[M + H]^+^	328
29	13.45	Caffeoyl tyrosine	n.a.	[M – H]-	342
				[M + H]^+^	344
30	23.17	Caffeoyl phenylalanine	n.a.	[M – H]^−^	326
				[M + H]^+^	328
31	1.72	Valine	Val	[M + H]^+^	118
32	2.46	Leucine or Isoleucine	Leu ou Ile	[M + H]^+^	132
33	2.78	Phenylalanine	Phe	[M + H]^+^	166
34	1.50	γ-Aminobutyric acid	GABA	[M + H]^+^	104
35	3.64	Tryptophan	Trp	[M + H]^+^	205
36	13.24	Tyrosine	Tyr	[M – H]^−^	180
37	1.56	Hexoses	n.a.	[M – H]^−^	179
38	1.56	Sucrose	n.a.	[M – H]^−^	341
				[M + Na]^+^	365
				[M + K]^+^	381
39	30.49	Atractyloside I	ATR-I	[M – H]^−^	727
40	24.84	Carboxyatractyloside I	CarATR-I	[M – H]^−^	771
41	14.89	Carboxyatractyloside II	CarATR-II	[M – H]^−^	525
42	16.59	Atractyloside II	ATR-II	[M – H]^−^	481
43	27.49	Carboxyatractyloside III (1)	CarATR-III_1	[M – H]^−^	609
44	31.2	Carboxyatractyloside III (2)	CarATR-III_2	[M – H]^−^	609
45	28.68	Carboxyatractyloside III (3)	CarATR-III_3	[M – H]^−^	609
46	32.32	Carboxyatractyloside III (4)	CarATR-III_4	[M – H]^−^	609
47	20.72	Atractyligenin	ATG	[M – H]^−^	319
48	13.24	Cumaric acid	n.a.	[M – H]^−^	163
49	1.60	d-Gluconic acid	n.a.	[M – H]^−^	195
50	3.22	Hydroxymethoxybenzoic acid (1)	n.a.	[M – H]^−^	167
51	12.27	Hydroxymethoxybenzoic acid (2)	n.a.	[M – H]^−^	167
52	23.44	Hydroxymethoxybenzoic acid (3)	n.a.	[M – H]^−^	167
53	1.66	Malic acid	n.a.	[M – H]^−^	133
54	2.63	Salicylic acid	n.a.	[M – H]^−^	137
55	26.42	Caffeoylvaleroylquinic acid	n.a.	[M – H]^−^	437
56	6.40	Caffeine	n.a.	[M + H]^+^	195
57	1.56	Trigonelline	n.a.	[M + H]^+^	138
58	27.49	Quercetin 3*-O-*glucoside	n.a.	[M – H]^−^	463
59	10.13	5*-O-*Caffeoylshikimic acid	5-CSA	[M – H]^−^	335
60	10.39	4*-O-*Caffeoylshikimic acid	4-CSA	[M – H]^−^	335

a n.a. - not applicable.

The elution order of compounds in the chromatographic analysis of coffee follows a pattern influenced by its polarity and structural complexity. Highly polar compounds, such as quinic acid, hexoses, and sucrose, exhibit short retention times due to their weak interactions with the C18 stationary phase.

In contrast, less polar compounds, including chlorogenic acids and atractylosides, demonstrate longer retention times owing to their increased hydrophobicity, which is attributed to the presence of additional phenolic groups or diterpenoid moieties in their structures. Moreover, chlorogenic acid isomers, such as 5-CQA, 4-CQA, and 3-CQA, were distinguished based on their retention times on the C18 column. Despite sharing the same molecular formula, these isomers differ in the positions of the caffeoyl groups attached to the quinic acid moiety, resulting in variations in their interaction with the stationary phase.

### 3.1 Chlorogenic Acid Derivatives (Phenylpropanoid Derivatives)

A total of 23 chlorogenic acids were identified in ESI(−) mode, Table 1 (ID 2-24). In general, the MS^2^ fragmentation spectra of quinic acid derivatives exhibit characteristic ions at *m*/*z* 173 and 191. The ester substituents (*trans*-cinnamic moiety) can be distinguished by specific ions: *m*/*z* 179 and 135 for caffeic acid; *m*/*z* 163 and 119 for *p*-coumaric acid; *m*/*z* 223 and 193 for sinapic acid; and *m*/*z* 207 and 163 for dimethoxycinnamic acid (Tables S1 and S2). These identified ions are consistent with those reported in the literature, using a similar type of equipment (ion trap) and fragmentation energy (CID 35%).^5,13^

Acil-quinic acids, commonly known as chlorogenic acids (CGAs), are esters formed between quinic acid and *trans*-cinnamic acids such as caffeic, ferulic, and coumaric acids. These compounds are produced by many plants, including fruits, vegetables, and herbs. Coffee is a rich source of these compounds, where more than 70 different chlorogenic acid compounds have been described in the literature on coffee.^6^

CGAs are characteristic components of coffee beans, where isomers of caffeoylquinic acids (CQAs), *p*-coumaroylquinic acids (*p*CoQAs), feruloylquinic acids (FQAs), dicaffeoylquinic acids (diCQAs), and caffeoylferuloylquinic acids (CFQAs) have already been reported. In coffee, esterification typically occurs at the 3, 4, and 5 positions of the quinic acid moiety, resulting in several combinations of these compounds.^5^

The nomenclature of chlorogenic acids follows the IUPAC standard for cyclitols, including quinic acid derivatives.^27^ According to this definition, the name is based on the hydroxycinnamic acids present in the structure. For instance, if caffeic acid is part of the molecule, it is referred to as a caffeoylquinic type of chlorogenic acid. However, due to the presence of different isomers, it is necessary to specify the position where the cinnamic acid derivative is attached to the quinic acid. Therefore, the nomenclature follows the pattern: position*-O-* + ester substituent + “quinic acid”. The most common substituent names are caffeoyl, *p*-coumaroyl, sinapoyl, feruloyl, and dimethoxycinnamoyl.^5,6^

Phenolic compounds, including chlorogenic acids, are considered bioactive and associated with a range of health benefits. These compounds possess antioxidant properties that enable them to neutralize free radicals within the body. This activity helps to mitigate cellular damage and reduce the risk of chronic diseases, such as cancer and cardiovascular disorders.^28^

During the roasting process, chlorogenic acids undergo degradation through ester bond cleavage. This reaction produces quinic acid derivatives, followed by hydrolysis and decarboxylation pathways, producing hydroquinone, phenol, and catechol derivatives. Alternative pathways involve the cinnamic acid moiety, yielding 2-methoxyphenol, 2-methoxy-4-methylphenol, 2-methoxy-4-vinylphenol, and 2-methoxy-4-ethylphenol, particularly from caffeoylquinic acid.^29^

From a sensory perspective, these volatile compounds contribute to negative attributes, such as smoky, spicy, medicinal, or leathery notes, and positive attributes, including floral, balsamic, sweet, and vanilla-like aromas, depending on their concentration.^30,31^

#### 3.1.1 Monoacylchlorogenic Acids (mCQA)

The MS^2^ experiments (Figure S1a–c) allow differentiating, in ESI(−) mode, the isomers in their [M – H]^−^ forms of *p*-coumaroylquinic acid (*p*CQA, *m*/*z* 337), caffeoylquinic acid (CQA, *m*/*z* 353), and feruloylquinic acid (FQA, *m*/*z* 367). Based on the available literature, compound identification was performed using ESI(−) data. All data for chlorogenic acids presented in this paper follow the IUPAC numbering system.^27^ Additionally, previously published data have been consulted to ensure consistency.^5,6,13,19^

The structures were also analyzed and identified in positive ionization mode (Figure S2a,b), [M + H]^+^, but isomer assignment was inconclusive with MS^2^, as MS^3^ could not be obtained for all compounds due to low ion counts. In MS^2^, CQA isomers exhibit analogous fragmentation profiles (base peak at *m*/*z* 163); however, in MS^3^, the 5*-O-*caffeoylquinic acid isomer can be distinguished by the intensity of the peak at *m*/*z* 163, while the 4-*O* and 3-*O* isomers display base peaks at *m*/*z* 145, with identical profiles, Figure S2a. The 4-*O* position can be differentiated by the *m*/*z* 285 signal in MS^2^, which is absent in the other fragmentation spectra. Figure S2c presents a proposed fragmentation mechanism for the positive ionization mode.

The detailed analysis of fragmentation profiles (Figure S1) and mechanisms (Figure 2) reveals a consistent pattern of the losses observed for monoacyl chlorogenic acids. Route A demonstrates a sequential loss of specific fragments, beginning with the cleavage of the ester bond. This process is facilitated by an intramolecular hydrogen rearrangement that releases the caffeoyl group as a neutral fragment (−C_9_H_6_O_3_), forming the ion at *m*/*z* 191, which corresponds to the quinic acid moiety. Subsequently, the *m*/*z* 191 → 173 transition indicates the elimination of a water molecule (−18 Da) from quinic acid.^32^

**Figure 2:**
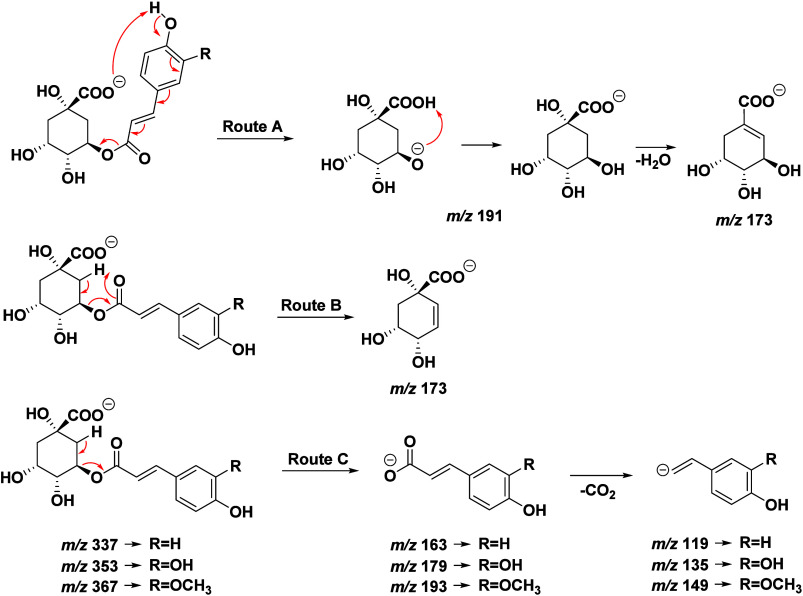
Proposed fragmentation mechanism for monoacylchlorogenic acids in ESI(−).

The computational investigation aimed to elucidate the proton transfer mechanism of Route A, which involves the transfer of a phenolic hydrogen to a carbonyl group. The proton transfer is of particular interest as it involves two functional groups that are relatively distant from each other in the molecular structure. To simulate this process, several computational steps were undertaken, including conformational analysis of the reactant and product molecules, transition state (TS) optimization, and Hessian matrix calculations.

The Gibbs free energy of the reactant, transition state, and product molecules were, respectively, −1296.04989, −1296.04775, and −1296.05149 hartree. From these results, Figure 3, it can be inferred that the Gibbs free energy of the product is slightly more negative than that of the reactant and transition state. This indicates that the proton transfer is a spontaneous process, as the product has a lower Gibbs free energy than both the reactant and the transition state, which suggests that the reaction is thermodynamically favorable.

**Figure 3:**
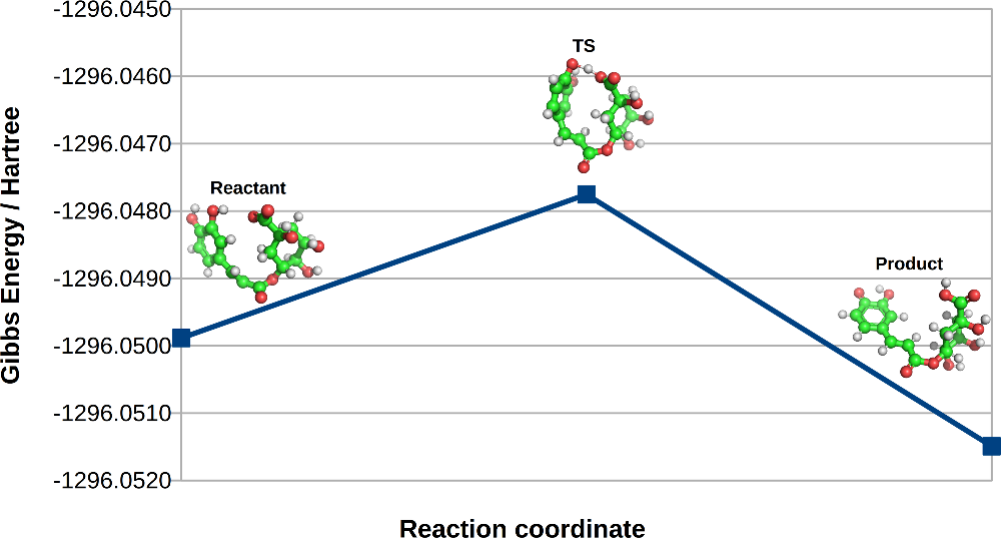
Gibbs free energy of reactant, transition state (TS) and product calculated using the ORCA5.04 package.

Further analysis of the transition state was conducted through a saddle point calculation, which yielded a single imaginary frequency of 213.36 i cm^–1^. The presence of this imaginary frequency is a clear indicator of a first-order saddle point, confirming that the structure represents a true transition state along the reaction pathway. The negative frequency corresponds to the mode of the proton transfer, reinforcing the notion that the system undergoes a shift from reactant to product through a transition state, where the proton moves from the phenolic group to the carbonyl oxygen.

In summary, the computational results support the hypothesis that the proton transfer from phenolic hydrogen to carbonyl oxygen is thermodynamically spontaneous and involves a well-defined transition state with a single imaginary frequency, confirming the mechanistic feasibility of the proposed reaction.

Route B involves a McLafferty rearrangement, which directly forms the ion at *m*/*z* 173 without passing through the intermediate at *m*/*z* 191. This pathway provides an alternative and efficient fragmentation mechanism.^32^ Route C involves abstraction of a hydrogen atom from the carbon α by the carboxylate (COO^–^) group. The electron rearrangement that follows forms a double bond (C=C) at the quinic acid core and breaks the bond between the quinic acid and the caffeoyl group. This process leads to the release of the caffeoyl group as a negatively charged fragment, while the quinic acid core remains as a neutral fragment. These proposed pathways highlight the critical role of eletronic stabilization and the influence of electronic and positional effects in the fragmentation mechanisms of monoacyl chlorogenic acids.

It is observed that all examined chlorogenic acids exhibit similar fragmentation patterns, suggesting a common origin derived from quinic acid, as shown in Figure 2. The differentiation of ester substituents is possible due to the cleavage of the bond between the cinnamic acid derivative and quinic acid (route C), leading to ions specific to the substituent. For example, the *m*/*z* 179 and 135 ions are characteristic of caffeic acid, while *m*/*z* 193 and 146 ions originate from ferulic acid. In this case, the second ion of each acid is produced from the neutral loss of CO_2_ (−44 Da).

The diagnostic signals, distinct for each ester substituent, are acceptable to confirm the identification of the compounds, as the signals and their respective relative intensities are consistent with MS^2^ experiments described in the literature under the same experimental conditions.^5,6^ Furthermore, it was possible to identify the isomers linked to quinic acid at the 5-*O*, 4-*O*, and 3-*O* positions for *p*-coumaroylquinic acid (*m*/*z* 337), caffeoylquinic acid (*m*/*z* 353), and feruloylquinic acid (*m*/*z* 367), as the isomers linked at the 5-*O* position exhibit higher stability of the fragment ion *m*/*z* 191. In comparison, those linked at the 4-*O* position have a higher stability of the *m*/*z* 173 ion. Table S1 presents the MS^2^ fragments of the identified monoacyl chlorogenic acids.

#### 3.1.2 Diacylchlorogenic Acids (dCQA)

The diCQA acids exhibited similar fragmentation behavior, leading to the precursor ion [diCQA-H]^−^, as observed in Figure 4 and Table S2. All identified compounds have a loss of the ester substituent (e.g., caffeic, ferulic, or coumaric acid), producing the [diCQA-cinnamoyl-H]^−^ ion as the base peak in MS^2^. However, these fragments have the same masses as the precursor ions of CQA, FQA, and *p*CQA, making it clear that the MS^n^ fragmentation profiles of these ions, produced from diCQA, are identical to those produced from mCQA (Figure S1).

**Figure 4:**
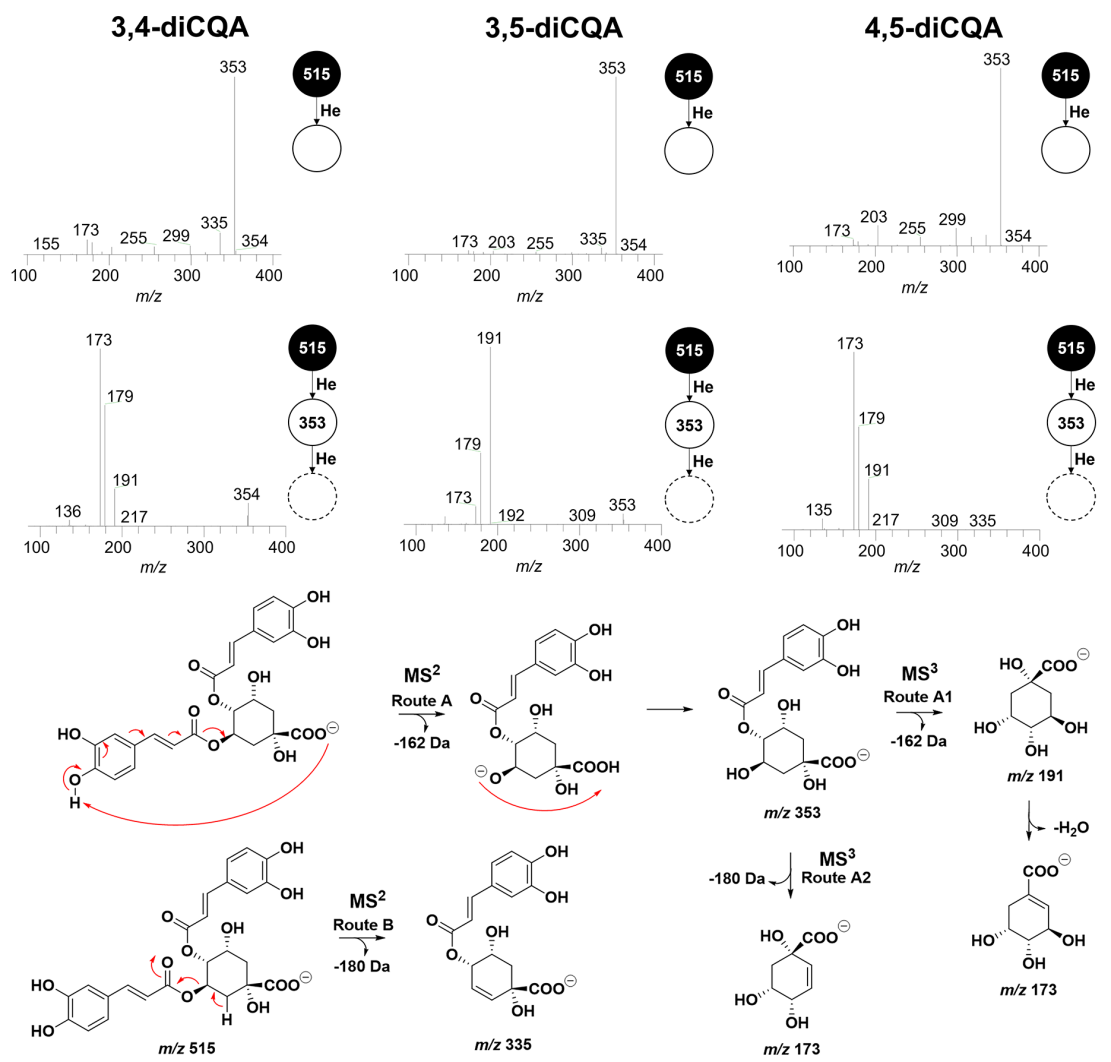
MS^2^ and MS^3^ fragmentation spectra and proposed fragmentation mechanism of diacylchlorogenic acids.

The compounds 3,4-diCQA and 4,5-diCQA exhibit a base peak at *m*/*z* 173 in MS^3^, while 3,5-diCQA is distinguished by a base peak at *m*/*z* 191 in MS^3^ (Figure 4). As mentioned earlier, due to the identical MS^2^ fragments of diCQA with the precursor ions of mCQA, the subsequent MS^3^ fragmentation of such fragments results in profiles analogous to those observed in the MS^2^ spectra in Figure 2. Therefore, the structure of the base peaks in MS^2^ for diCQA is defined, making it possible to assume which part of the chlorogenic acid was lost.

Given the base peak at *m*/*z* 173 in the MS^3^ spectrum, characteristic of the 4-*O* position isomers, the *m*/*z* 353 signal in MS^2^ for 3,4-diCQA and 4,5-diCQA can be attributed to [4-CQA-H]^−^, while, for 3,5-diCQA, the base peak in MS^2^ can be attributed to [3-CQA-H]^−^. Therefore, the removal of cinnamic acid from the 5-*O* position is less probable. Table S2 presents the MS^2^ and MS^3^ fragments of the identified diacyl chlorogenic acids.

The diCQAs also include the combination of two different cinnamic acids (Figures S3 and S4), such as 3*-O-*caffeoyl-4*-O-*feruloylquinic acid, 3*-O-p*-coumaroyl-4*-O-*caffeoylquinic acid, 3-*O*-dimethoxycinnamoyl-5-*O*-caffeoylquinic acid, or 4-*O*-sinapoyl-3-*O*-caffeoylquinic acid. All identified compounds exhibited a base peak at *m*/*z* 353 ([CQA-H]^−^), 367 ([FQA-H]^−^), 381 ([dimethoxycinnamoylquinic acid-H]^−^), or 397 ([sinapoylquinic acid-H]^−^) in MS^2^. The presence of *m*/*z* 353 as the base peak in MS^2^ indicates the loss of ferulic acid, which, combined with the base peak at *m*/*z* 191 in MS^3^, clearly defines that the compound examined lacks substitution at the 4 or 5 position, thus identifying it as 3F-5CQA.

For 4Si-3CQA and 3Si-5CQA (*m*/*z* 559), the *m*/*z* 559 → 397 transition in MS^2^ indicates the loss of the caffeic acid moiety (Figure S3). In MS^3^, the fragmentation profile for *m*/*z* 397, producing a base peak at 193, indicates that the sinapic acid is linked at the 3-*O* position of the quinic acid moiety, therefore 3Si-5CQA. The transition *m*/*z* 397 → 223 indicates that sinapic acid is linked at the 4-*O* position (4Si-3CQA).^6^

For 3D-5CQA (*m*/*z* 543) and 4D-5FQA (*m*/*z* 557), the *m*/*z* 543 → 381 and *m*/*z* 557 → 381 transitions in MS^2^ indicate the loss of the caffeic and ferulic acid moieties, respectively (Figure S3). In MS^3^, the fragmentation profile for *m*/*z* 381, from *m*/*z* 543, produces a base peak at *m*/*z* 207, which indicates that the dimethoxycinnamic acid is linked at the 3-*O* position of the quinic acid moiety (3D-5CQA). The transition *m*/*z* 381 → 173, from *m*/*z* 559, indicates that dimethoxycinnamic acid is linked at the 4-*O* position (4D-5FQA).^5,6^

For 3-*p*Co-4CQA (*m*/*z* 499), the *m*/*z* 499 → 353 transition in MS^2^ indicates the loss of the *p*-coumaric acid moiety, Figure S4. In MS^3^, a fragmentation profile for *m*/*z* 353 is observed, identical to that of 4-CQA, while the presence of *m*/*z* 337 ([*p*CoQA-H]^−^) in MS^2^ indicates that the acid is linked at the 3 position.^5,13^

### 3.2 Cinnamoyl–Amino Acid Conjugates

Green coffee beans are characterized by their significant content of cinnamic acid conjugates. The most well-known are the conjugates with quinic acid, known as chlorogenic acids.^5,6,13^ In addition to these, cinnamic acids can also be conjugated with amino acids such as caffeoyl tryptophan and *p*-coumaroyl tyrosine. These compounds were ionized and characterized in ESI­(+) and ESI(−) modes, as shown in Table S3. Due to the low intensity of the signals, it was not possible to obtain MS^3^ spectra for all compounds; however, MS^2^ was sufficient for confirmation in ESI­(+) and ESI(−) modes.

In ESI(−)­MS^2^, the signal at *m*/*z* 229 for tryptophan conjugates and *m*/*z* 206 for tyrosine, Table S3, corresponds to the loss of a vinylphenol fragment characteristic of the cinnamic acid residue, i.e., −136 Da for caffeic acid, −120 Da for *p*-coumaric acid, and −150 Da for ferulic acid. The fragmentation of these signals involves deamidation, producing base peaks in MS^3^ at *m*/*z* ∼185 and ∼163, as illustrated in Figure 5. On the other hand, in ESI­(+)­MS^2^, the presence of base peaks at *m*/*z* 147, 163, and 177 indicates residues related to *p*-coumaric, caffeic, and ferulic acids, respectively.^20^

**Figure 5:**
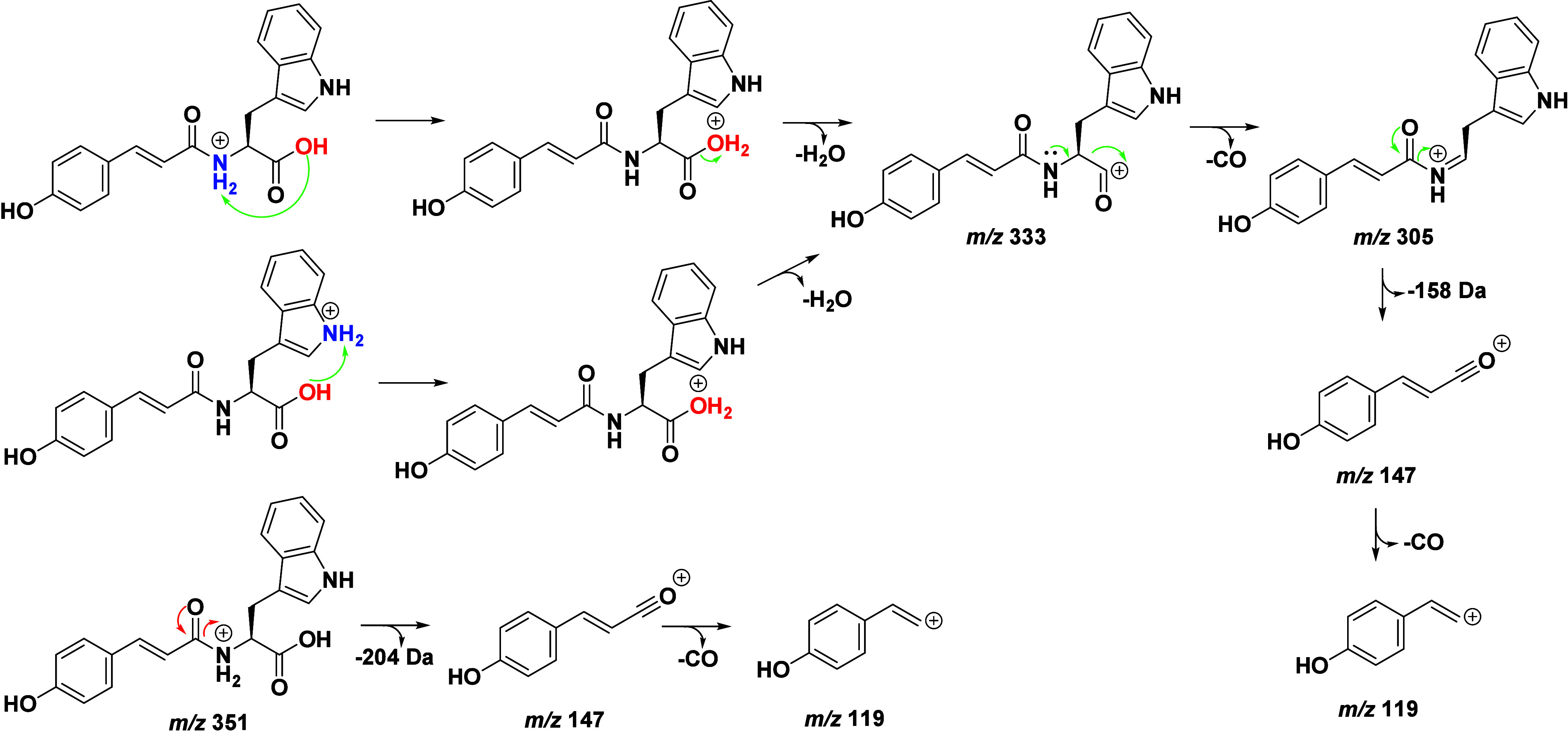
Proposal for the fragmentation of cinnamoylamides in ESI­(+).

### 3.3 Amino Acids

Amino acids are composed of amine (NH_2_) and carboxyl (COOH) functional groups attached to a side chain that characterizes the compound. They play an important role in the Maillard reaction, which occurs during the roasting process by condensation of a reducing sugar with an amino group. This reaction produces volatile compounds such as furans, furanones, and pyranones, which impart desirable sensory attributes including caramel, buttery, bready, and sweet notes. Furthermore, α-dicarbonyl compounds are produced during this process, acting as precursors for other compound classes.^31,33,34^

Simultaneously, Strecker degradation involves the interaction of α-dicarbonyl compounds with an amino acid, producing pyrazines, aldehydes, and carbon dioxide. Pyrazines, in particular, are essential to the sensory profile of coffee, contributing attributes such as nutty, hazelnut, fruity, floral, earthy, roasted, and peanut-like notes. These compounds are among the key volatiles responsible for the distinct coffee’s aroma.^31,33^

The characterization of amino acids was performed only in positive mode as no signals related to them were observed in negative mode (except for tyrosine). Additionally, due to the low intensity of the signals, MS^3^ spectra could not be obtained for all compounds. However, MS^2^ was acceptable for confirmation based on the literature and the NIST library.^31,35–37^ In total, five compounds were identified in their protonated form ([M + H]^+^). The MS^2^ fragmentation profiles for these compounds, Table S4, show that the common fragment among amino acids is the immonium ion ([M + H – H_2_O – CO]^+^), formed by the sequential loss of H_2_O and CO.

The loss of water is derived from the transfer of a proton attached to the nitrogen to the adjacent hydroxyl group (Figure 6). This is followed by a loss of 18 Da from the inductive cleavage of −OH_2_. The acylium ion formed is unstable and consequently undergoes a neutral loss of CO (−28 Da), providing the immonium ion, which has a higher stability due to its resonance capability. Therefore, the [M + H – H_2_O – CO]^+^ fragment is known as a diagnostic ion for several amino acids.^35,36^ Due to the stability of the immonium ion, MS^3^ experiments are required. Based on the profiles, the neutral loss of NH_3_ (−17 Da) is observed via inductive cleavage, producing the [M + H – H_2_O – CO – NH_3_]^+^ ion.

**Figure 6:**
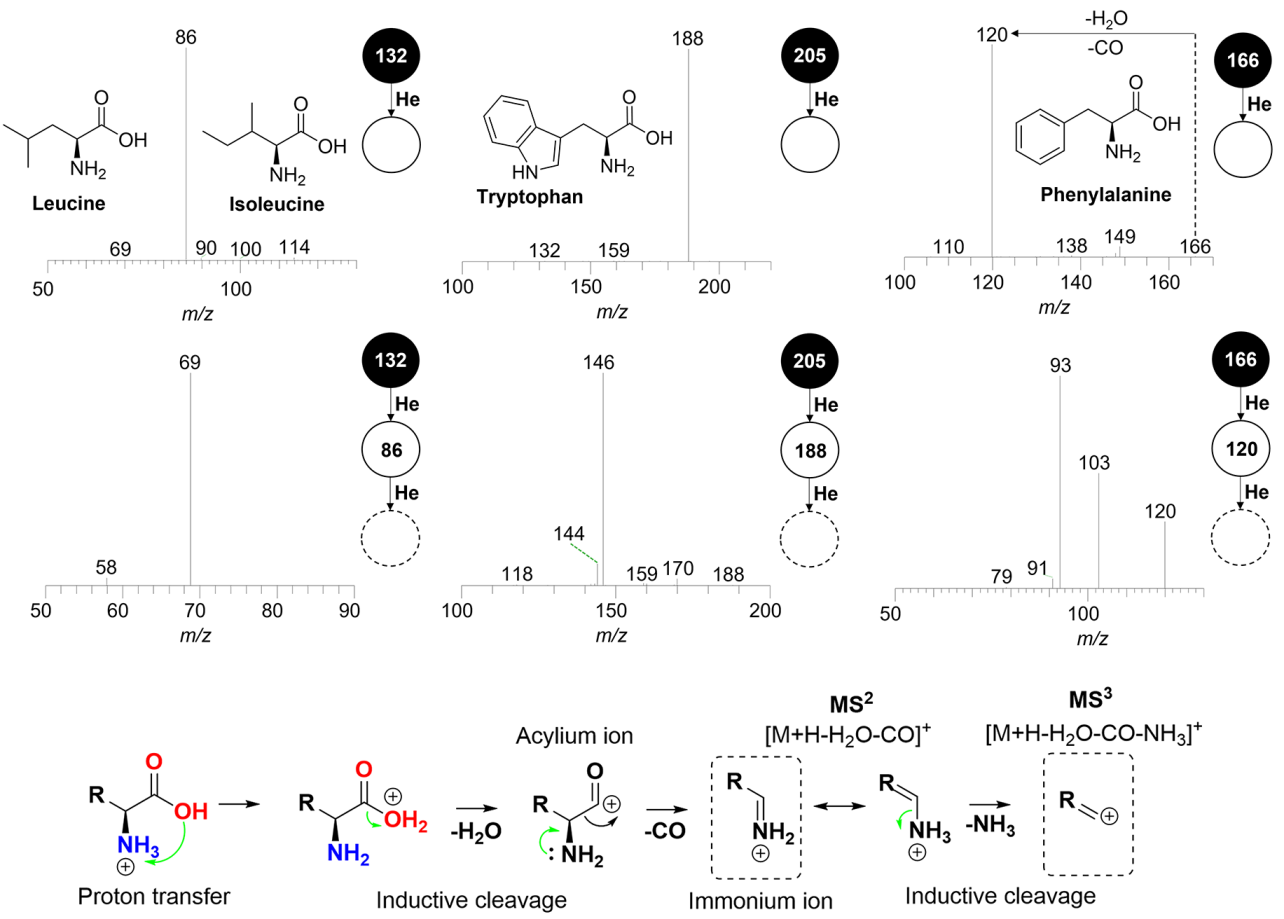
Profile and mechanisms of MS^2^ and MS^3^ fragmentation of the identified amino acids in ESI­(+).

As summarized in Table S4, the identified amino acids exhibited fragmentation profiles analogous to the generic mechanism previously illustrated, with losses of 46 Da (loss of H_2_O and CO) in the transition from the precursor ion to the base peak in MS^2^ and 17 Da in the transition from the MS^2^ base peak to MS^3^.

### 3.4 Carbohydrates

Carbohydrates constitute approximately 50% of the dry composition of green coffee. These compounds play an essential role in the production of volatile compounds by a series of complex transformations including the Maillard reaction. This reaction involves the condensation of a reducing sugar with an amino compound, producing a wide variety of compounds responsible for the characteristic flavor of roasted coffee.^31,34^

Only two carbohydrates were identified, i.e., sucrose (or another isomer) and a hexose. The hexose can be attributed to the *m*/*z* 179 signal ([M – H]^−^) which can be associated with glucose or fructose, both present in coffee. As shown in Figure S5, MS^2^ fragmentation of the ion in question can follow different pathways. The first consists of the *m*/*z* 179 → 161 → 143 → 113 transitions, which can be associated with two successive losses of 18 Da (−H_2_O), followed by the neutral loss of CH_2_O (−30 Da). Another alternative is the initial loss of CH_2_O, leading to the formation of the *m*/*z* 149 ion, which can follow two pathways: the loss of H_2_O or CH_2_O, corresponding to the transitions *m*/*z* 149 → 131 and *m*/*z* 149 → 119, respectively.^38^

Sucrose can be observed in positive and negative modes as [M – H]^−^, [M + Na]^+^, and [M + K]^+^ ions. The identification of sucrose (Figure S6) was based on the MS^2^ fragments of the *m*/*z* 341 ion ([M – H]^−^). The base peak at *m*/*z* 179 indicates cleavage of the glycosidic bond associated with [glucose-H]^−^ or [fructose-H]^−^. The subsequent fragments are a result of successive transitions *m*/*z* 179 → 161 → 143 → 113 and *m*/*z* 179 → 149 → 119, indicating losses of H_2_O and CH_2_O, as previously discussed for glucose. Additionally, the identity of the compounds was confirmed by comparison with that of the NIST library for the fragmentations of *m*/*z* 365 and *m*/*z* 341.

The assignment of *m*/*z* 381 ([M + K]^+^) as sucrose was made based on the identical retention time relative to other ions, as well as the same mass loss in the transitions *m*/*z* 365 → 203 and *m*/*z* 381 → 219 (−162 Da) and the 180 Da loss in the transitions *m*/*z* 365 → 185 and *m*/*z* 381 → 201. In addition, the 18 Da difference between the two most intense signals in both MS^2^ spectra (*m*/*z* 203 → 185 and *m*/*z* 219 → 201) can be associated with the neutral loss of H_2_O.

### 3.5 Atractylosides

Atractylosides are glycosylated diterpenes found in a wide variety of natural products, particularly in coffee, where the considerable differences in their content between *Coffea arabica* and *Coffea canephora* var. robusta led to the proposal of using these compounds for botanical authenticity control.^39^

The primary mechanism of action associated with atractyloside derivatives involves the disruption of mitochondrial oxidative phosphorylation by the inhibition of adenosine nucleotide transfer. This mechanism is responsible for their toxicity in humans, potentially leading to acute hepatic or kidney injury.^40,41^ However, further investigation is required to elucidate critical aspects, particularly regarding biologically safe exposure limits, the behavior of these compounds during the roasting process, and their contribution to sensory profile.^42^

Table S5 presents the fragments and their respective intensities from the MS^2^ and MS^3^ experiments for the nine atractylosides identified. In summary, MS^3^ was required only for the carboxylated compounds as in MS^2^ they only lost CO_2_.

For carboxyatractyloside I (*m*/*z* 771, [M – H]^−^), as shown in Figure 7, the *m*/*z* 771 → 727 transition indicates a neutral loss of 44 Da in MS^2^, corresponding to the decarboxylation (−CO_2_). Subsequently, in MS^3^, the *m*/*z* 727 → 643 transition corresponds to the loss of the 3-methylbutylaldehyde group (−84 Da, −C_5_H_8_O). Meanwhile, the *m*/*z* 727 ion undergoes a 162 Da loss (−C_6_H_10_O_5_), corresponding to cleavage of the glycosidic bond. The *m*/*z* 565 → 481 transition also corresponds to the loss of 3-methylbutylaldehyde, while the *m*/*z* 481 → 319 transition corresponds to the loss of −C_6_H_10_O_5_ (−162 Da). The *m*/*z* 643 → 625 and *m*/*z* 481 → 463 transitions correspond to the neutral loss of water.^39,42^

**Figure 7:**
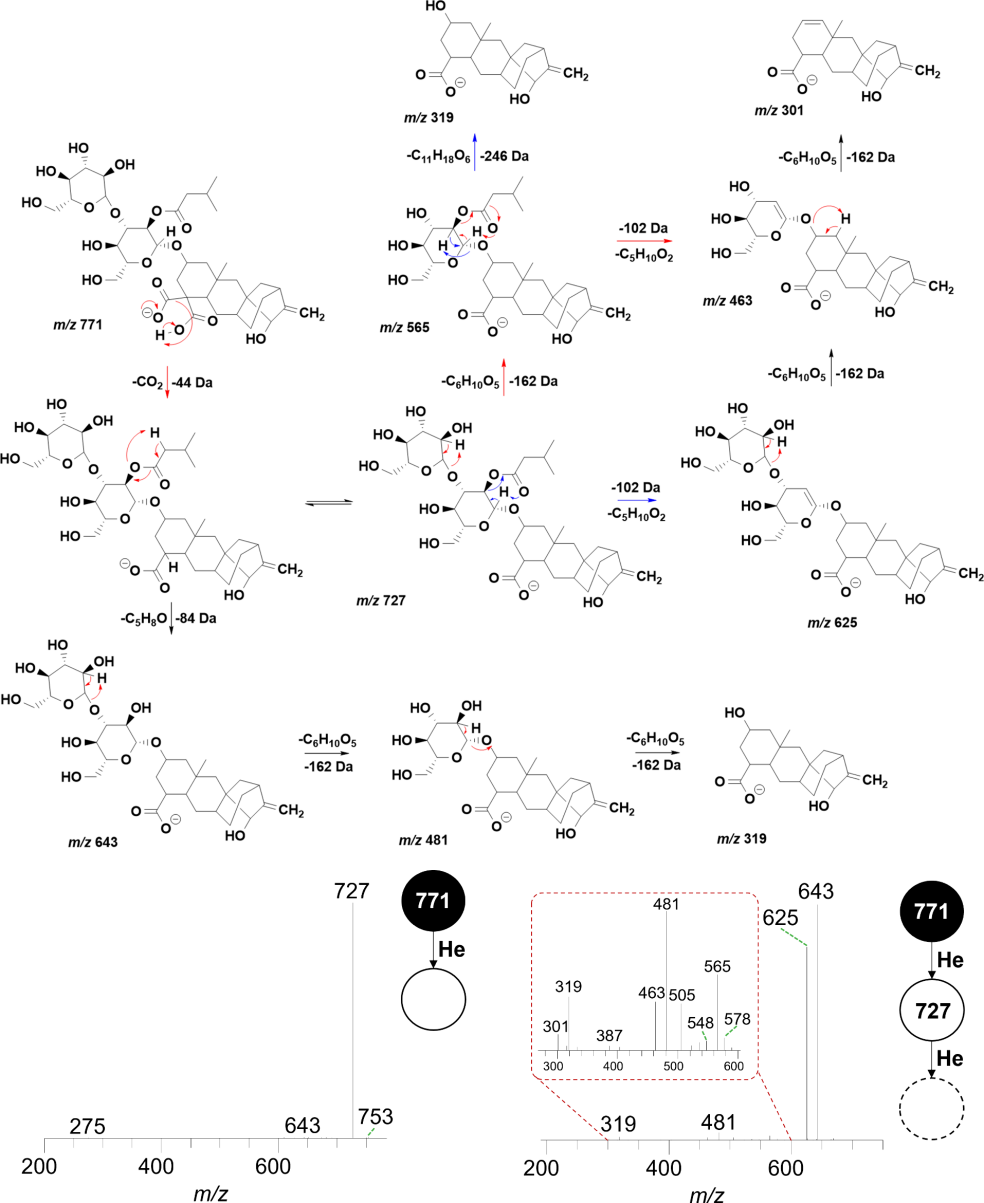
Mechanism and fragmentation profile of carboxytractyloside I (*m*/*z* 771).

An analogous behavior can be observed for atractyloside I (Figure S7), except for the initial loss of CO_2_. A difference in retention time was noted between the two compounds, which may be associated with the presence of the carboxyl group in the carboxyatractyloside I structure, which can increase its polarity and, consequently, a shorter retention time (a difference of ∼5 min between the compounds). For carboxyatractyloside II (*m*/*z* 525), in Figure S8, a notable similarity with the fragmentation profile of carboxyatractyloside I is observed. The main distinction between these two structures lies in the absence of a hexose moiety.

In carboxyatractyloside II, the *m*/*z* 319 → 301 (−18 Da; −H_2_O) and *m*/*z* 301 → 257 transitions (−44 Da; −CO_2_) are more evident in this compound. Similarly, the MS^2^ fragmentation profile of atractyloside II is identical to the MS^3^ profile (*m*/*z* 525 → 481) of the *m*/*z* 481 ion, as they correspond to the same structure. The difference between the two molecules lies in the presence of a carboxyl group, in which first fragmentation (MS^2^) involves CO_2_ elimination.

Four isomers of CarATR-III were found (Figure S9), where an initial loss of CO_2_ was observed in MS^2^. In MS^3^, the *m*/*z* 565 → 481 transition corresponds to the loss of the 3-methylbutylaldehyde group (−84 Da, −C_5_H_8_O), the *m*/*z* 481 → 463 to the loss of −H_2_O, and finally the *m*/*z* 481 → 301 to the loss of a hexose (−180 Da). However, it is observed that all of the isomers exhibit similar fragmentation profiles, making the available analytical data insufficient to provide detailed structures for each isomer. Therefore, the 3-methylbutylaldehyde group (R–COCH_2_CH­(CH_3_)_2_) can be attached at four different positions on the hexose.

Finally, atractyligenin (*m*/*z* 319) exhibits two main transitions, *m*/*z* 319 → 275 and 319 → 273, corresponding to the neutral loss of CO_2_ and formic acid (−46 Da, HCOOH), respectively, followed by possible water losses (*m*/*z* 275 → 257, 273 → 255, and 257 → 239).^42^

### 3.6 Other Compounds

For quinic acid (*m*/*z* 191, [M – H]^−^), as shown in Figure S10a, the *m*/*z* 191 → 127 transition is observed, resulting from the successive loss of formic acid (HCOOH, −46 Da) and water (H_2_O, −18 Da). Additionally, the main transition at *m*/*z* 191 → 127 results from the loss of water from quinic acid. The *m*/*z* 111 ion is the result of the successive loss of 44 Da (CO_2_; *m*/*z* 173 → 129) and 18 Da (H_2_O) from the *m*/*z* 129 fragment.^43^

Caffeoylvaleroylquinic acid was identified based on the proposed fragmentation mechanisms. In Figure S10b, the base peak at *m*/*z* 275 results from the neutral loss of the caffeic acid moiety (−162 Da, C_9_H_6_O_2_). Subsequently, the *m*/*z* 275 → 173 transition occurs due to the loss of valeric acid (−102 Da, C_5_H_10_O_2_). An additional plausible pathway is the initial loss of valeric acid (*m*/*z* 437 → 335), followed by the cleavage of caffeic acid also resulting in the *m*/*z* 173 ion. Table S6 presents the other compounds identified as [M – H]^−^ ion and their MS^2^ fragments. For *p*-coumaric acid (*m*/*z* 163), only the *m*/*z* 119 signal was observed in MS^2^ (neutral loss of CO_2_).

For gluconic acid, naturally produced from glucose, it is suggested that the signals correspond to the *m*/*z* 195 → 177 → 159 transition from successive losses of water. The origin of the *m*/*z* 129 ion could not be explained; however, the fragmentation profile is similar to that found in the literature.^44^

Three hydroxy-methoxybenzoic acid derivatives (Figure S11) were identified; however, based on the available analytical information, it was not possible to elucidate the isomers among the six structures already described for coffee.^45^ The MS^2^ profile shows the following transitions: *m*/*z* 167 → 152, from the loss of CH_3_ (−15 Da); *m*/*z* 167 → 149, from the loss of H_2_O; and *m*/*z* 167 → 123 → 108, corresponding to the consecutive loss of CO_2_ and CH_3_. For malic acid, the *m*/*z* 133 → 115 transition corresponds to the neutral loss of water, while, for salicylic acid, the *m*/*z* 137 → 93 transition is due to the neutral loss of CO_2_.

Caffeoylshikimic acids (CSAs) are distinguished from quinic acid derivatives due to the absence of a hydroxyl group (−OH). As a result, the compounds linked to the caffeic acid moiety exhibit an ion at *m*/*z* 335 in ESI(−). According to Figure S12, CSAs have a base peak in MS^2^ at *m*/*z* 179 ([caffeic acid-H]^−^), indicating the neutral loss of the shikimic acid moiety. Additionally, the *m*/*z* 335 → 173 transition reveals the neutral loss of −162 Da (−C_9_H_6_O_3_), while the *m*/*z* 335 → 155 transition (−180 Da; −C_9_H_8_O_4_) is related to the elimination of caffeic acid and a hydroxyl group from shikimic acid. The *m*/*z* 335 → 173 transition indicates the loss of the shikimic acid moiety (162 Da), followed by the decarboxylation of caffeic acid (−44 Da; *m*/*z* 179 → 135).

However, due to the similarity in the fragmentation patterns of the observed isomers, distinguishing between them was not feasible based solely on the mass spectra. Therefore, it was necessary to rely on the information provided by chromatographic separation. According to the literature, compounds linked at the 5-position (5-CSA) elute earlier compared to those linked at the 4-position (4-CSA).^6^

For trigonelline (Figure S13), the *m*/*z* 138 → 110 and *m*/*z* 138 → 94 transitions correspond to the neutral loss of CO (−28 Da) and CO_2_ (−44 Da), respectively. Trigonelline is an alkaloid that, after the roasting process, plays an important role in the formation of coffee’s aroma and flavor. This compound has been extensively investigated for its potential in cardiovascular disease prevention and its anticancer properties. Moreover, its capacity to regulate enzymes involved in glucose and lipid metabolism supports its role in reducing blood sugar and cholesterol levels.^46^

For caffeine, the *m*/*z* 195 → 138 transition corresponds to the neutral loss of C_2_H_3_NO (−57 Da) due to a retro-Diels-Alder mechanism. The consecutive transition (*m*/*z* 138 → 110) results from the neutral loss of CO. Other important transitions are *m*/*z* 195 → 163 (−32 Da, CH_3_OH) and *m*/*z* 195 → 151 (−44 Da, CH_3_CHO). In the MS^2^ spectrum of caffeine, the most intense signal at *m*/*z* 138 indicates higher ion stability due to the resonance stabilization of this structure.^47,48^

Finally, for quercetin 3*-O-*glucoside (Figure S14), a compound belonging to the flavonoid class, an initial loss of 18 Da (−H_2_O) from the glycosidic moiety is proposed, corresponding to the elimination of water, followed by the loss of 144 Da (−C_6_H_8_O_4_). Another possible pathway is the direct loss of the glycosidic portion (−162 Da, C_6_H_10_O_5_).^44^

## 4 Conclusions

The present results conclusively demonstrated the efficacy and potential of liquid chromatography coupled with low-resolution mass spectrometry (LC-ESI(±)­LTQ MS) for compound identification. Based on sequential fragmentation experiments, literature review, and proposed fragmentation mechanisms, it was possible to identify 60 compounds, providing a comprehensive view of the complex metabolomic composition of coffee. This study not only consolidates the analytical approach used but also offers new possibilities for future research in food chemistry, particularly offering a detailed characterization of metabolites, reference spectra, and fragmentation pathways.

## Supplementary Material



## Notes

Table S1. Fragments in ESI(−)­MS^2^ of monoacyl chlorogenic acids; Table S2. Fragments in ESI(−)­MS^2^ and MS^3^ of diacyl chlorogenic acids; Table S3. Fragments in ESI(±)­MS^2^ and MS^3^ of cinnamoyl–amino acid conjugates; Table S4. Fragments in ESI(±)­MS^2^ and MS^3^ of identified amino acids; Table S5. Fragments in ESI(−)­MS^2^ and MS^3^ of identified atractylosides; Table S6. Fragments in ESI(−)­MS^2^ of organic acids; Figure S1. MS^2^ spectra of *p*CQA, CQA, and FQA acids in ESI(−) mode; Figure S2. MS^2^ and MS^3^ mass spectra of CQA acid and FQA acid and fragmentation mechanism of mCQA in ESI­(+) mode; Figure S3. MS^2^ and MS^3^ mass spectra of dimethoxycinnamoyl and sinapoyl derivatives of quinic acid in ESI(−) mode; Figure S4. ESI(−)­MS^2^ and MS^3^ spectra of some diCQA compounds; Figure S5. Mechanism and ESI(−)­MS^2^ fragmentation of glucose; Figure S6. MS^2^ spectra of the ions [M – H]^−^, [M + Na]^+^, and [M + K]^+^, related to sucrose; Figure S7. ESI(−)­MS^2^ mass spectrum of atractyloside I; Figure S8. Mechanism and ESI(−)­MS^2^ and MS^3^ fragmentation spectra of carboxytractyloside II; Figure S9. Mechanism and ESI(−)­MS^2^ and MS^3^ fragmentation spectrum of carboxytractyloside III_1-4; Figure S10. Mechanism and ESI(−)­MS^2^ fragmentation spectrum of caffeic acid and caffeoylvaleroylquinic acid; Figure S11. ESI(−)­MS^2^ mass spectra of hydroxymethoxybenzoic acid derivatives and their possible structures; Figure S12. Spectra and fragmentation mechanism of 5-CSA and 4-CSA in ESI(−) mode; Figure S13. ESI­(+)­MS^2^ mass spectrum and fragmentation mechanism of trigonelline and caffeine; Figure S14. ESI(−)­MS^2^ mass spectrum and fragmentation mechanism of quercetin 3-*O*-glucoside (PDF)
