# Supplementary material for: LC-ESI(±)-LTQ MSn‑Based Metabolomic Profiling of Coffee: Fragmentation Pathways for Identification of Major Polar Compounds
Source: J Am Soc Mass Spectrom. 2025 May 13;36(6):1213–26. doi: 10.1021/jasms.4c00418 (PMC12142669; doi:10.1021/jasms.4c00418)
Supplement: Supplementary file 1 [file js4c00418_si_001.pdf]

SUPPLEMENTARY MATERIAL

LC-ESI(±)-LTQ MS^n-Based Metabolomic Profiling of Coffee: Fragmentation Pathways for Identification of Major Polar Compounds

Marcos Valério Vieira Lyrio[a]*, Danieli Grancieri Debona[a], Amanda Eiriz Feu[a], Nayara Araujo dos Santos[a,b], Arlan da Silva Gonçalves[a,b], Ricardo Machado Kuster[a], Eustáquio Vinícius Ribeiro de Castro[a], and Wanderson Romão[a,b]*

[a]Federal University of Espírito Santo/UFES, Department of Chemistry, Campus Goiabeiras, Avenida Fernando Ferrari, 514, CEP 29075-910 Vitoria, Espírito Santo, Brazil

[b]Federal Institute of Espírito Santo – Campus Vila Velha, Av. Ministro Salgado Filho, Soteco, Vila Velha, Espírito Santo 29106-010, Brazil

*Corresponding authors: mrvaleriovieira@gmail.com and wanderson.romao@ifes.edu.br

Supplementary Material Captions:

Table S1. Fragments in ESI(-)MS^2 of monoacyl chlorogenic acids.
Table S2. Fragments in ESI(-)MS^2 and MS^3 of diacyl chlorogenic acids.
Table S3. Fragments in ESI(±)MS^2 and MS^3 of cinnamoyl-amino acid conjugates.
Table S4. Fragments in ESI(±)MS^2 and MS^3 of identified amino acids.
Table S5. Fragments in ESI(-)MS^2 and MS^3 of identified atractylosides.
Table S6. Fragments in ESI(-)MS^2 of organic acids.
Figure S1. MS^2 spectra of (A) *p*CQA, (B) CQA, and (C) FQA acids in ESI(-) mode.
Figure S2. MS^2 and MS^3 mass spectra of (A) CQA acid and (B) FQA acid; and (C) fragmentation mechanism of mCQA in ESI(+) mode.
Figure S3. MS^2 and MS^3 mass spectra of (A) dimethoxycinnamoyl and (B) sinapoyl derivatives of quinic acid in ESI(-) mode.
Figure S4. ESI(-)MS^2 and MS^3 spectra of some diCQA compounds.
Figure S5. Mechanism and ESI(-)MS^2 fragmentation of glucose.
Figure S6. MS^2 spectra of the ions $[M-H]^-$, $[M+Na]^+$, and $[M+K]^+$, related to sucrose.
Figure S7. ESI(-)MS^2 mass spectrum of atractyloside I.
Figure S8. Mechanism and ESI(-)MS^2 and MS^3 fragmentation spectra of carboxytractyloside II.
Figure S9. Mechanism and MS^2 and MS^3 fragmentation spectrum of carboxytractyloside III_1-4.
Figure S10. Mechanism and MS^2 fragmentation spectrum of (A) caffeic acid and (B) caffeoylvaleroylquinic acid.
Figure S11. ESI(-)MS^2 mass spectra of hydroxymethoxybenzoic acid derivatives and their possible structures.
Figure S12. Spectra and fragmentation mechanism of 5-CSA and 4-CSA in ESI(-) mode
Figure S13. ESI(+)MS^2 mass spectrum and fragmentation mechanism of trigonelline and caffeine.
Figure S14. ESI(-)MS^2 mass spectrum and fragmentation mechanism of quercetin 3-*O*-glucoside.

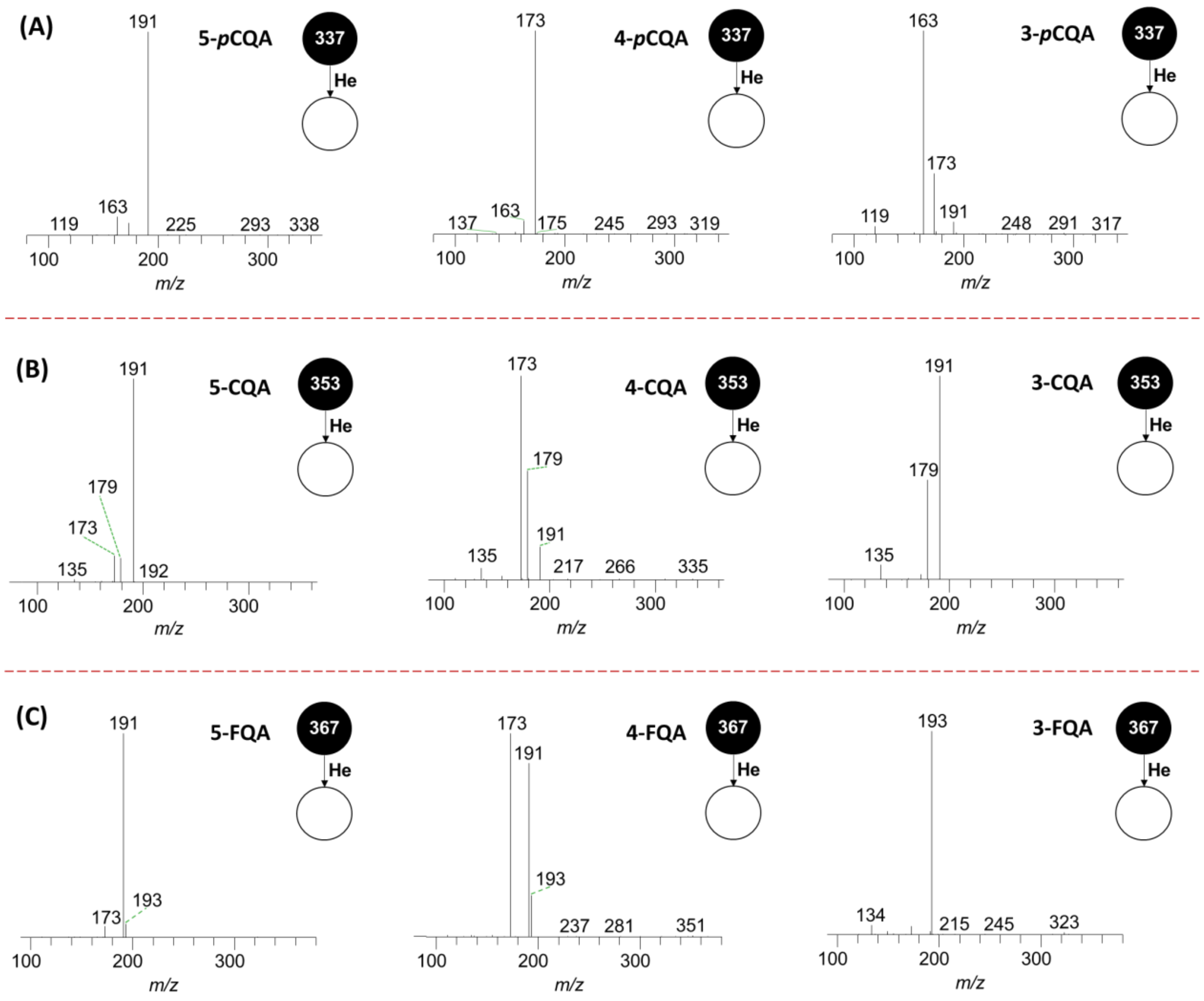

Figure S1. MS^2 spectra of (**A**) *p*CQA, (**B**) CQA, and (**C**) FQA acids in ESI(-) mode.

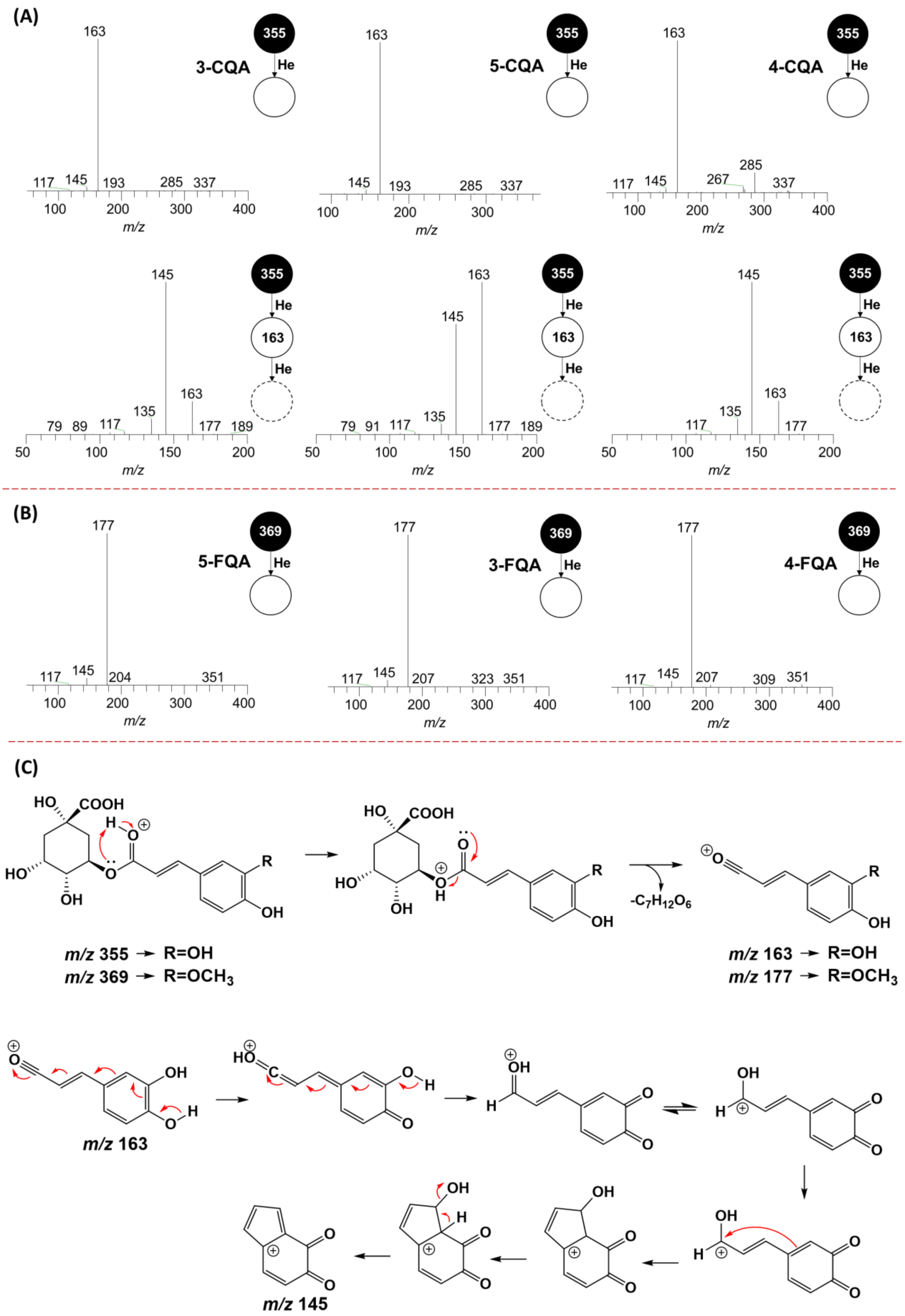

Figure S2. MS^2 and MS^3 mass spectra of **(A)** CQA acid and (**B**) FQA acid; and (**C**) fragmentation mechanism of mCQA in ESI(+) mode.

Table S1. Fragments in ESI(-)MS^2 of monoacyl chlorogenic acids

ID	Compound	*m/z*	MS^2 *m/z* (Relative intensity %)
2	5-*O*-caffeoylquinic acid	353	191(100), 173(9), 179(8)
3	4-*O*-caffeoylquinic acid	353	173(100), 179(53), 191(14)
4	3-*O*-caffeoylquinic acid	353	191(100), 179(49), 135(7)
5	5-*O*-feruloylquinic acid	367	191(100), 193(6), 173(5)
6	4-*O*-feruloylquinic acid	367	173(100), 191(85)
7	3-*O*-feruloylquinic acid	367	193(100), 134(4), 173(4), 149(2), 191(2)
8	5-*O*-p-coumaroylquinic acid	337	191(100), 163(7), 173(6)
9	4-*O*-p-coumaroylquinic acid	337	173(100), 163(6)
10	3-*O*-p-coumaroylquinic acid	337	163(100), 173(30), 191(6), 119(4)

Table S2. Fragments in ESI(-)MS^2 and MS^3 of diacyl chlorogenic acids

ID	Compound	*m/z*	MS^2	*m/z*	MS^3
11	3-O-dimethoxycinnamoyl-5-O-caffeoylquinic acid	543	381(100), 335(2)	381	207(100), 173(4)
12	4-O-dimethoxycinnamoyl-5-O-feruloylquinic acid	557	381(100), 349(41),173(9)	381	173(100), 207(16)
13	4-O-sinapoyl-3-O-caffeoylquinic acid	559	397(100), 173(3), 335(3)	397	173(100), 223(22)
14	3-O-sinapoyl-5-O-caffeoylquinic acid	559	397(100), 223(6), 335(3)	397	223(100)
15	4,5-di-*O*-caffeoylquinic acid	515	353(100), 203(11), 299(10)	353	173(100), 179(68), 191(21)
16	3,5-di-*O*-caffeoylquinic acid	515	353(100), 335(4), 173(3)	353	191(100), 179(40), 173(10)
17	3,4-di-*O*-caffeoylquinic acid	515	353(100), 335(12), 173(8)	353	173(100), 179(69), 191(35)
18	4,5-di-*O*-feruloylquinic acid	543	367(100), 349(24), 173(6)	367	173(100), 193(53)
19	3-*O*-caffeoyl-4-*O*-feruloylquinic acid	529	367(100), 335(20), 353(16), 173(13)	367	173(100), 193(27)
20	3-*O*-caffeoyl-5-*O*-feruloylquinic acid	529	353(100), 367(41), 335(4)	353	191(100), 179(56), 173(25)
21	4-*O*-feruloyl-5-*O*-caffeoylquinic acid	529	367(100), 353(84), 335(17), 173(11)	367	173(100), 193(50), 191(32), 134(2)
22	3-*O*-feruloyl-5-*O*-caffeoylquinic acid	529	367(100), 353(9), 335(4)	-	-
23	4-*O*-caffeoyl-5-*O*-feruloylquinic acid	529	353(100), 367(44), 335(7)	353	173(100), 179(59), 191(31), 135(6)
24	3-*O*-p-coumaroyl-4-*O*-caffeoylquinic acid	499	353(100), 337(68), 335(30), 319(13)	353	173(100), 179(71), 191(45), 135(7)

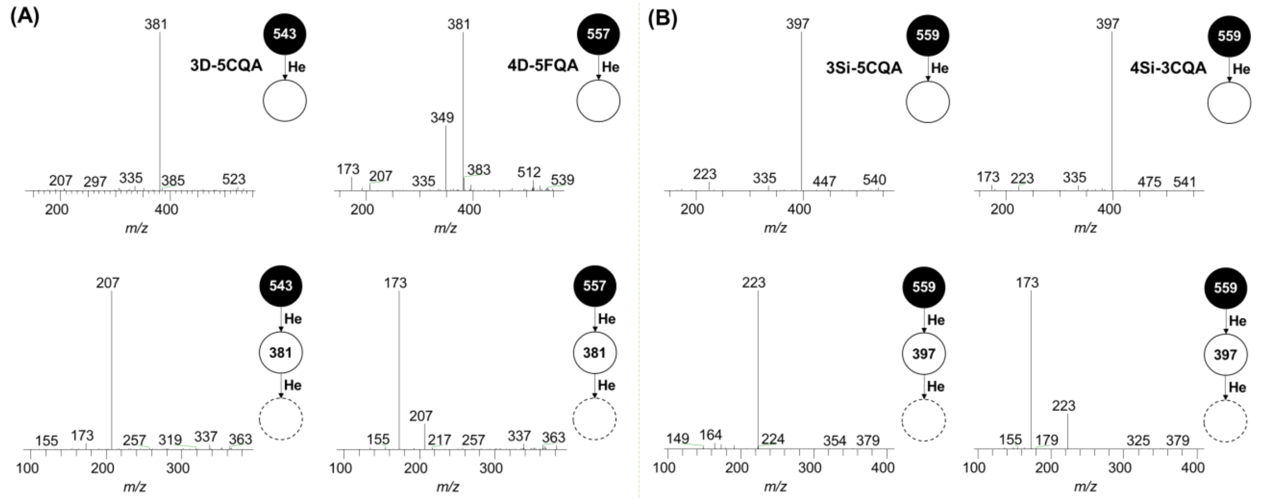

Figure S3. MS^2 and MS^3 mass spectra of (A) dimethoxycinnamoyl and (B) sinapoyl derivatives of quinic acid in ESI(-) mode.

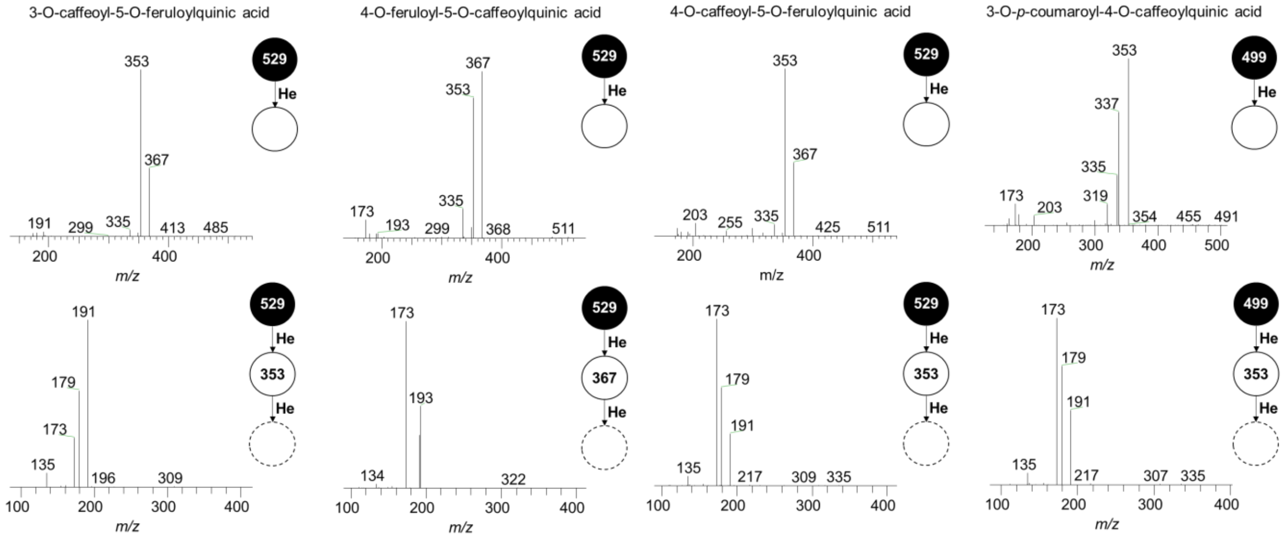

Figure S4. ESI(-)MS^2 and MS^3 spectra of some diCQA compounds.

Table S3. Fragments in ESI(±)MS^2 and MS^3 of cinnamoyl-amino acid conjugates

ID	Compound	Ion	*m/z*	MS^2	*m/z*	MS^3
25	*p*-Coumaroyl tryptophan	$[M-H]^-$	349	229(100), 193(12), 173(11)	229	185(100), 186(65), 100(25)
		$[M+H]^+$	351	147(100), 159(60), 333(13), 119(5)	-	-
26	Caffeoyl tryptophan	$[M-H]^-$	365	229(100), 135(5), 161(2)	229	185(100), 186(100), 100(26)
		$[M+H]^+$	367	163(100), 182(24), 136(8)	-	-
27	Feruloyl tryptophan	$[M-H]^-$	379	229(100), 186(6), 175(6), 185(4)	-	-
		$[M+H]^+$	381	177(100), 159(12), 145(10)	-	-
28	*p*-Coumaroyl tyrosine	$[M-H]^-$	326	282(100), 206(63), 147(11), 163(9)	206	163(100), 119(3), 100(3)
		$[M+H]^+$	328	147(100), 136(12), 119(3)	-	-
29	Caffeoyl tyrosine	$[M-H]^-$	342	206(100), 163(5), 135(3)	206	163(100), 119(3)
		$[M+H]^+$	344	163(100), 182(24), 136(7)	-	-
30	Caffeoyl phenylalanine	$[M-H]^-$	326	190(100), 135(10), 147(5), 161(3)	-	-
		$[M+H]^+$	328	163(100), 166(17), 120(7)	-	-

Table S4. Fragments in ESI(±)MS^2 and MS^3 of identified amino acids

ID	Compound	Ion	*m/z*	MS^2	*m/z*	MS^3
31	Valine	$[M+H]^+$	118	72(100), 100(1)		
32	Leucine or Isoleucine	$[M+H]^+$	132	86(100), 114(1)	86	69(100), 58(3)
33	Phenylalanine	$[M+H]^+$	166	120(100), 149(5), 148(1)	120	93(100), 103(54), 120(31), 91(5)
34	Gamma-aminobutyric acid	$[M+H]^+$	104	87(100), 86(36), 60(18)		
35	Tryptophan	$[M+H]^+$	205	188(100)	188	146(100), 144(10), 170(3)
36	Tyrosine	$[M-H]^-$	180	163(100), 136(12), 119(11), 93(11)		

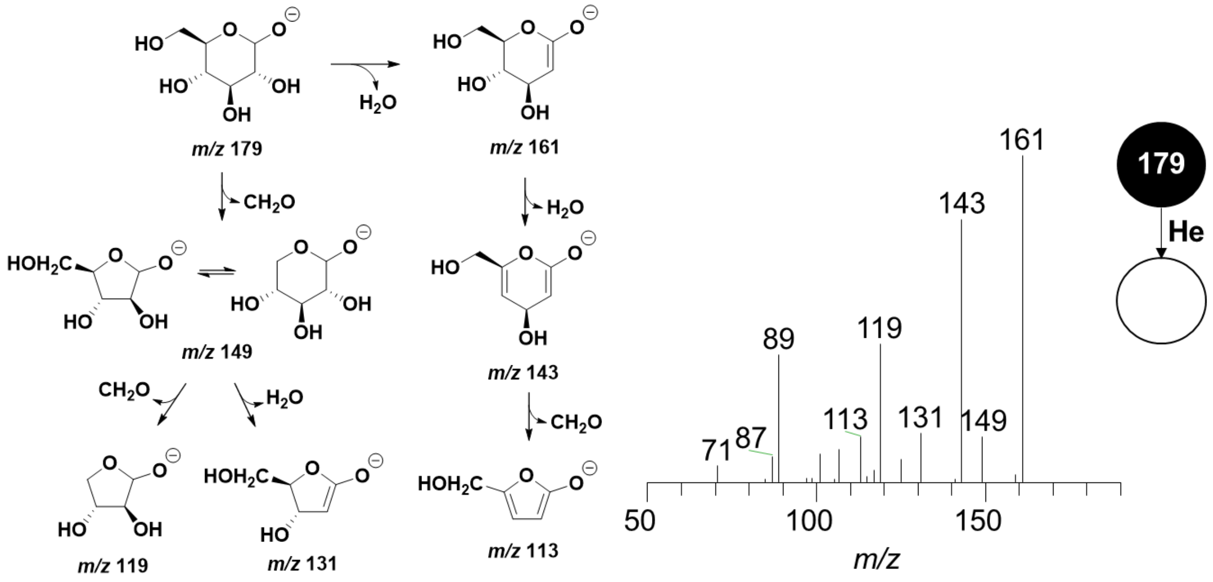

Figure S5. Mechanism and ESI(-)MS^2 fragmentation of glucose.

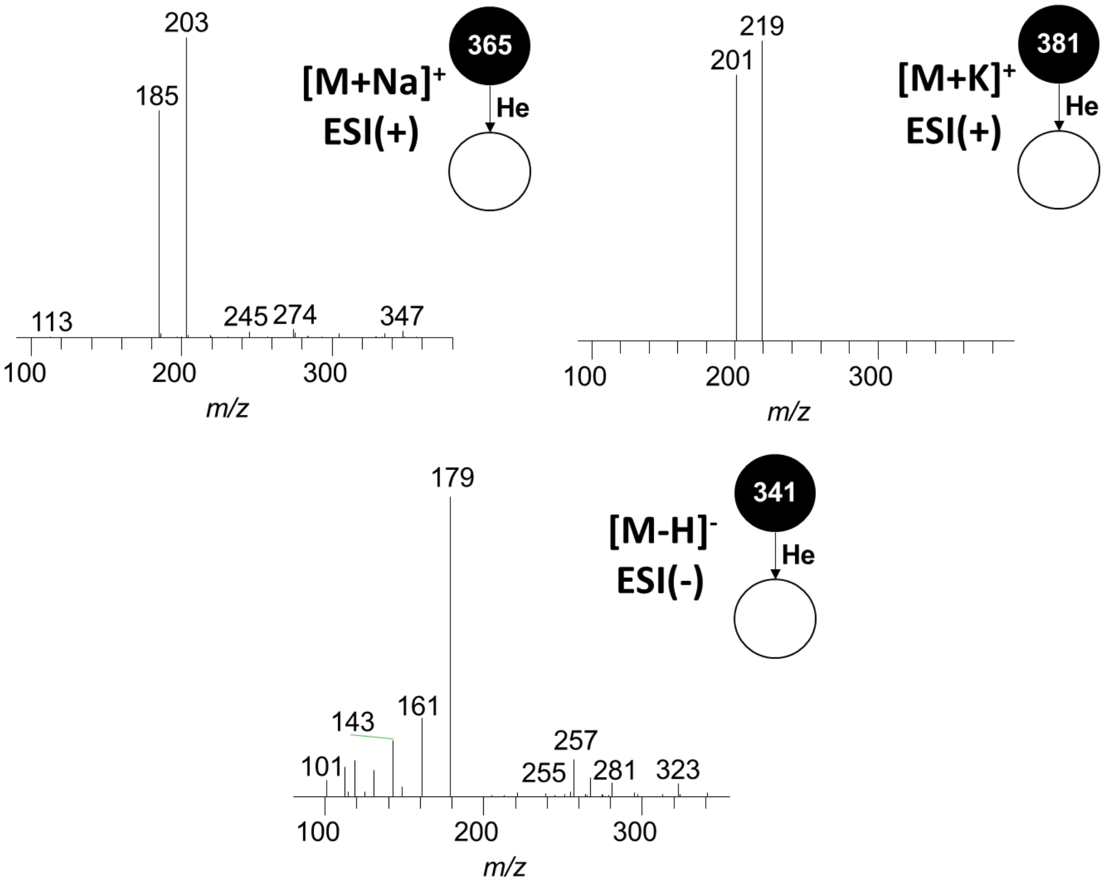

Figure S6. MS^2 spectra of the ions [M-H]$^-$, [M+Na]$^+$, and [M+K]$^+$, related to sucrose.

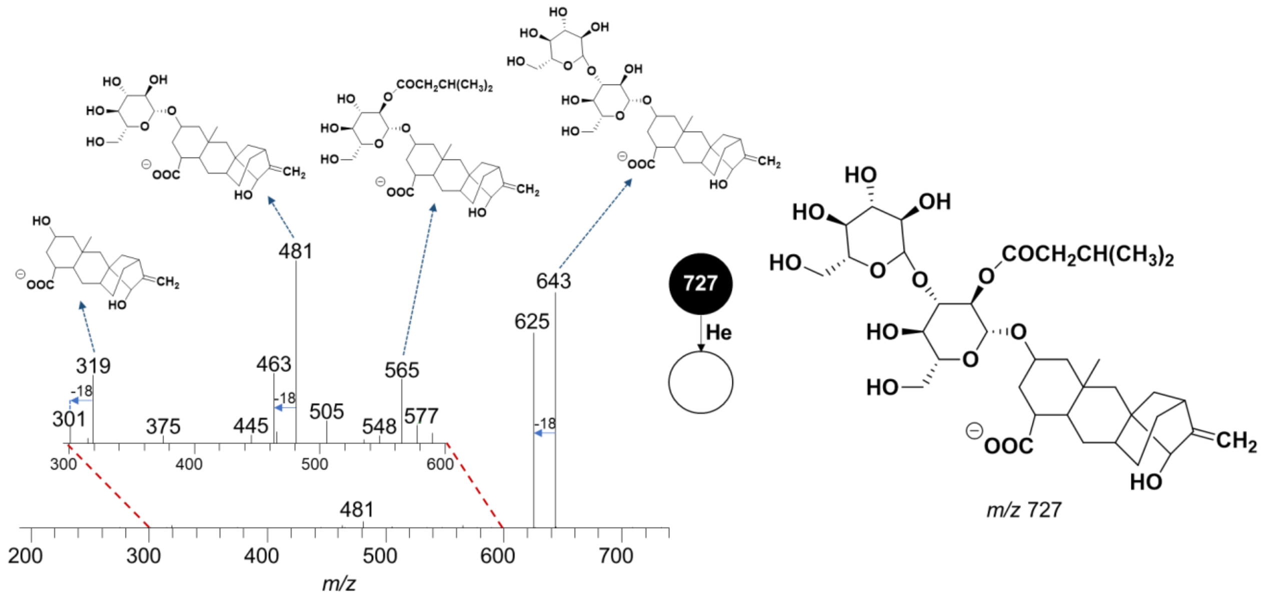

Figure S7. ESI(-)MS^2 mass spectrum of atractyloside I.

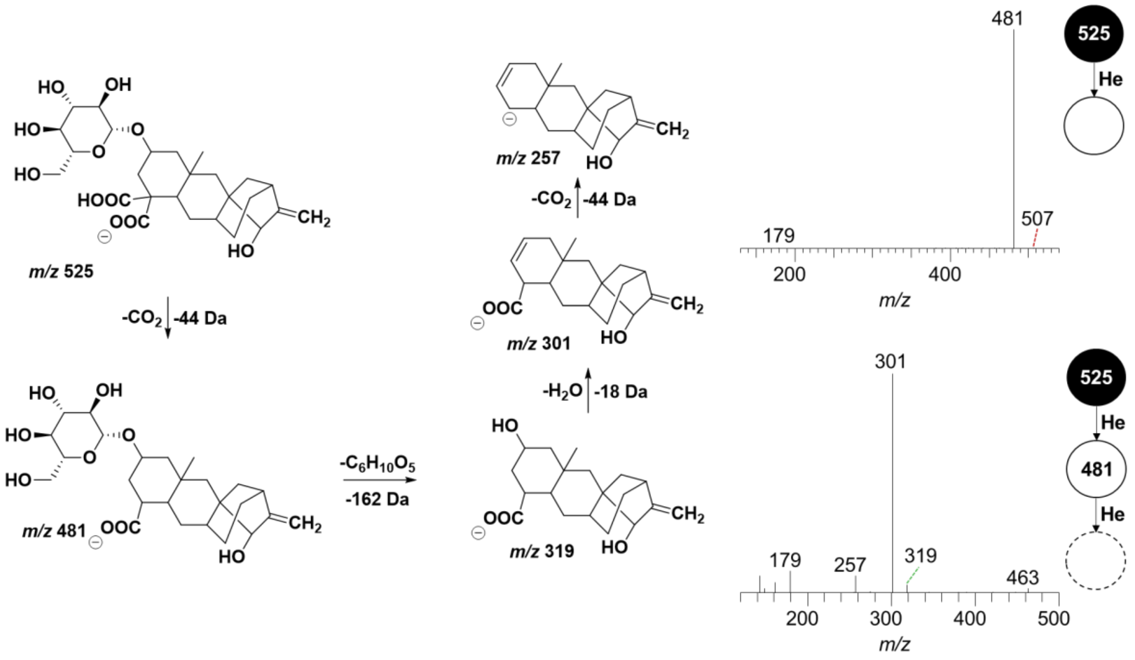

Figure S8. Mechanism and ESI(-)MS^2 and MS^3 fragmentation spectra of carboxytractyloside II.

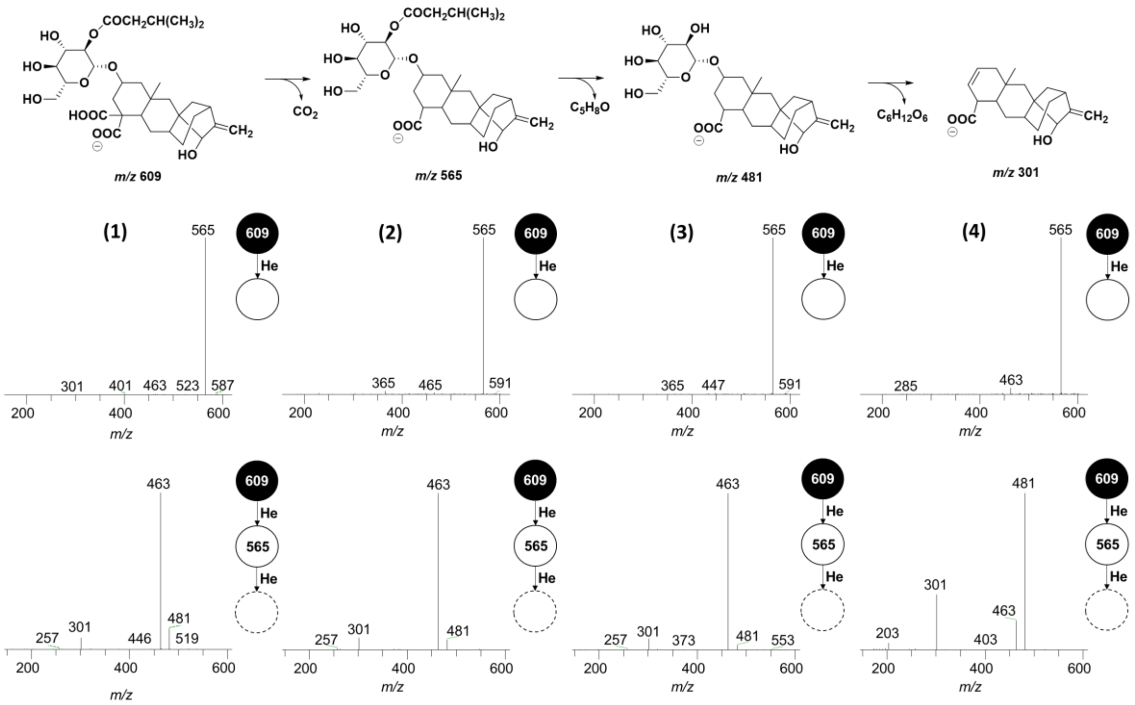

Figure S9. Mechanism and ESI(-)MS^2 and MS^3 fragmentation spectrum of carboxytractyloside III_1-4.

Table S5. Fragments in ESI(-)MS^2 and MS^3 of identified atractylosides

ID	Compound	*m/z*	MS^2	*m/z*	MS^3
39	Atractyloside I	727	643(100), 625(83), 481(3), 463(1)	-	-
40	Carboxyatractyloside I	771	727(100), 753(1)	727	643(100), 625(82), 481(3), 565(2)
41	Carboxyatractyloside II	525	481(100)	481	301(100), 179(10), 143(8), 257(8), 319(4)
42	Atractyloside II	481	301(100), 319(17), 437(14), 257(12)	-	-
43	Carboxyatractyloside III (1)	609	565(100)	565	463(100), 481(13), 301(7)
44	Carboxyatractyloside III (2)	609	565(100), 365(2)	565	463(100), 301(8), 481(7)
45	Carboxyatractyloside III (3)	609	565(100)	565	463(100), 301(7)
46	Carboxyatractyloside III (4)	609	565(100), 463(4)	565	481(100), 301(35), 463(19), 203(4)
47	Atractyligenin	319	275(100), 273(77), 276(31), 291(23), 274(14), 255(12)	-	-

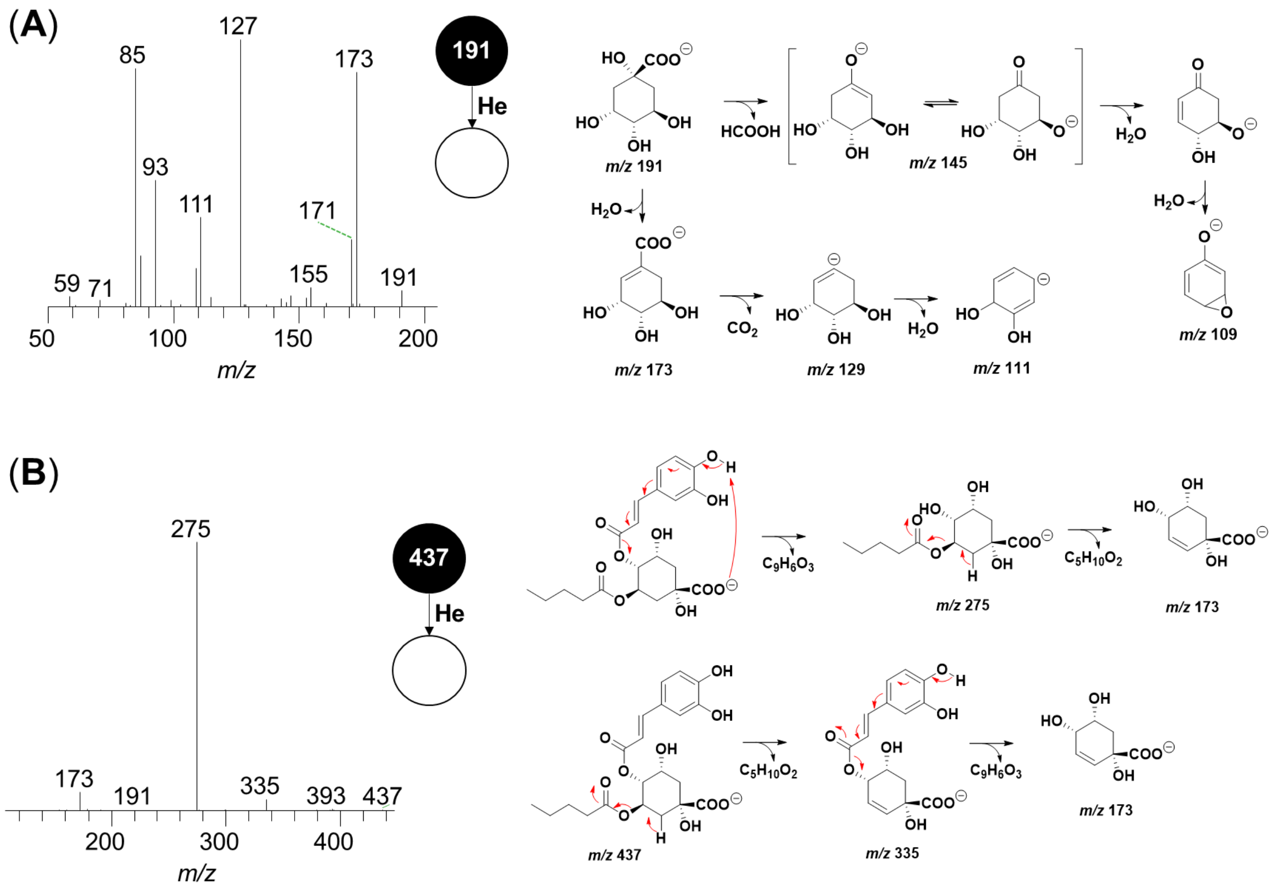

Figure S10. Mechanism and ESI(-)MS^2 fragmentation spectrum of **(A)** caffeic acid and **(B)** caffeoylvaleroylquinic acid.

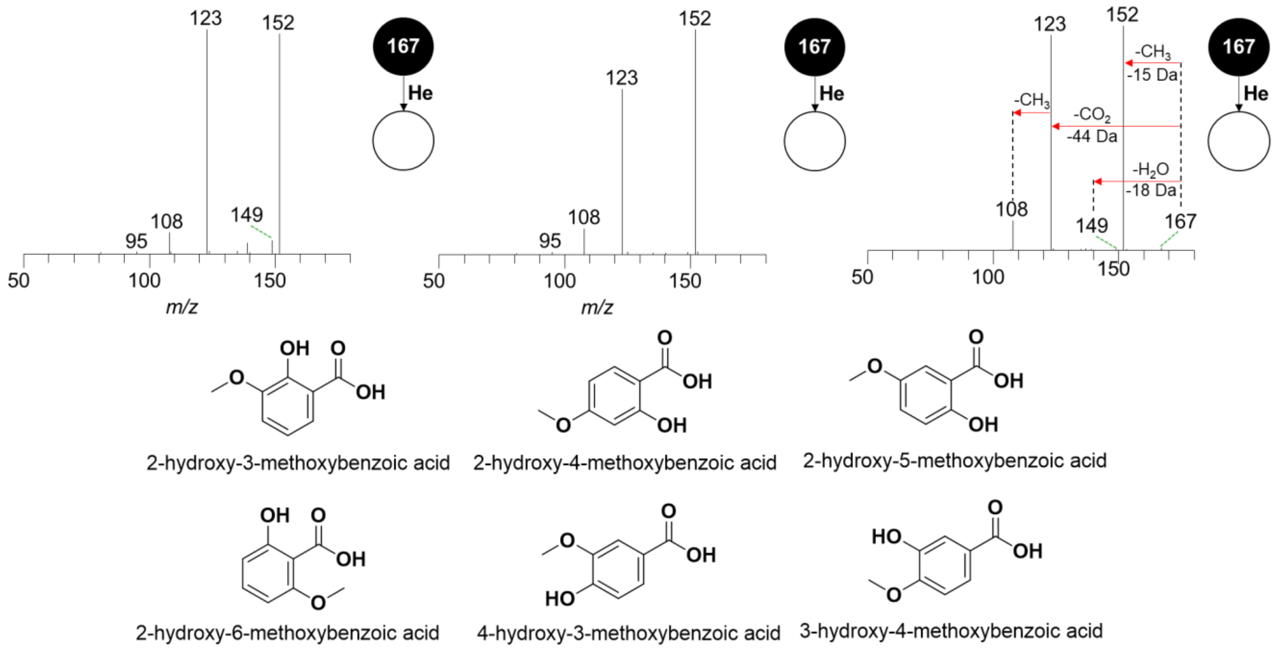

Figure S11. ESI(-)MS^2 mass spectra of hydroxymethoxybenzoic acid derivatives and their possible structures.

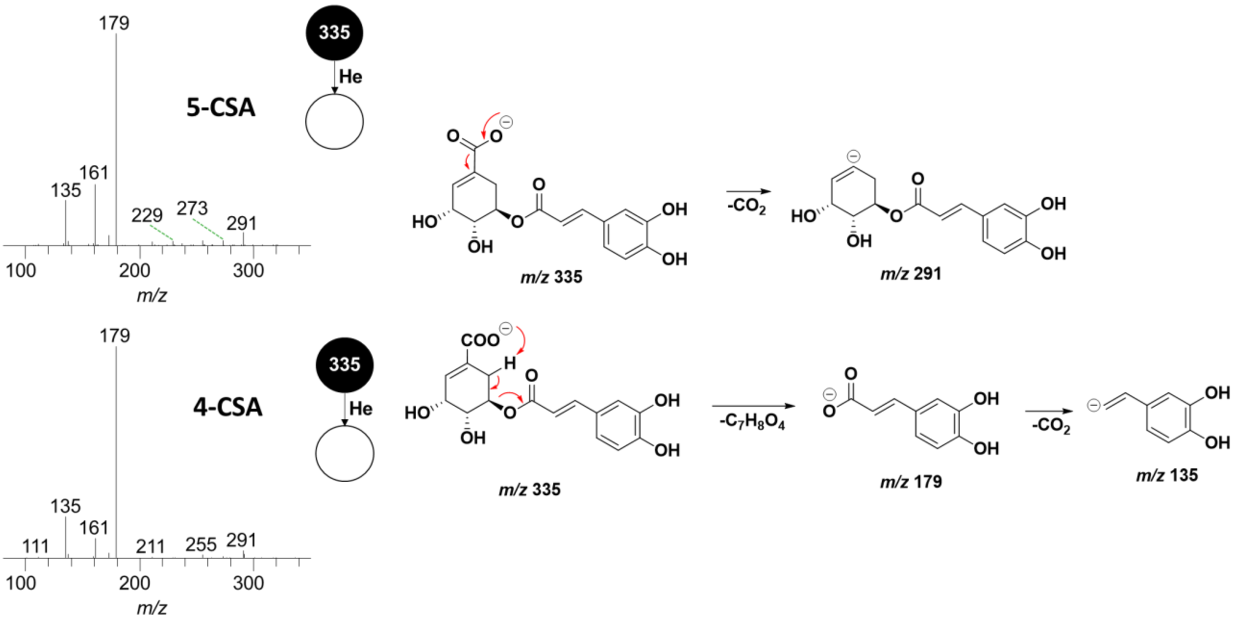

Figure S12. Spectra and fragmentation mechanism of 5-CSA and 4-CSA in ESI(-) mode.

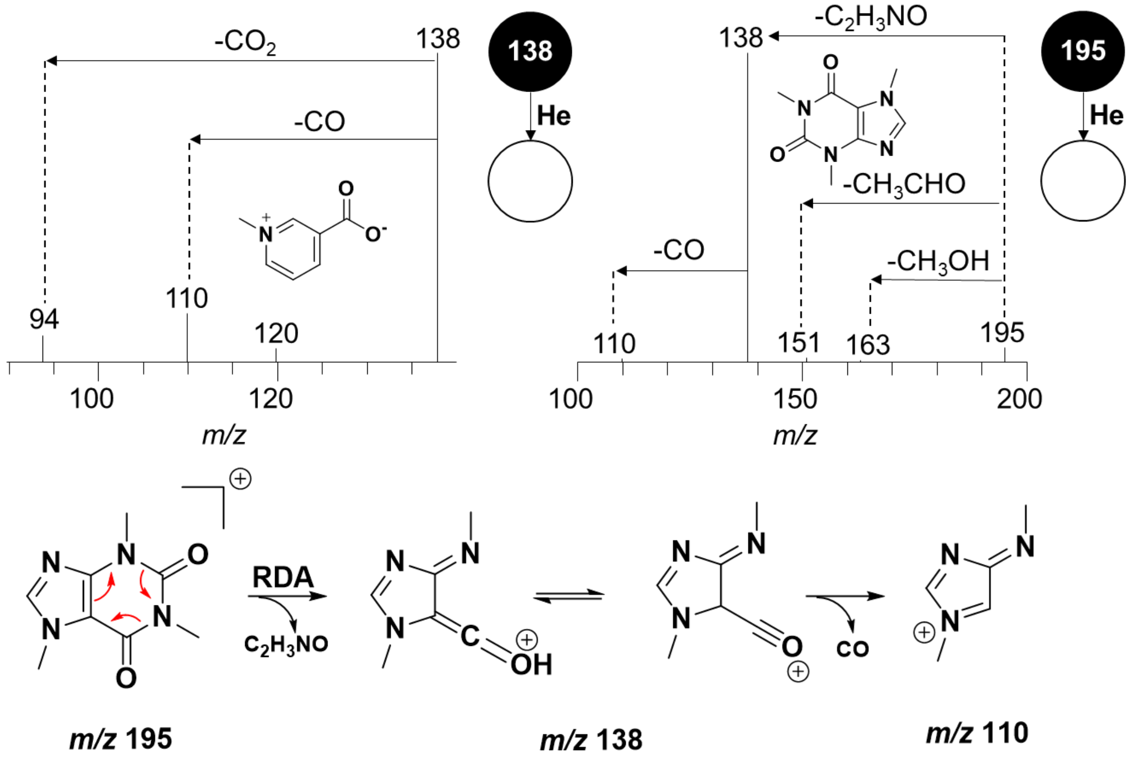

Figure S13. ESI(+)MS[2] mass spectrum and fragmentation mechanism of trigonelline and caffeine.

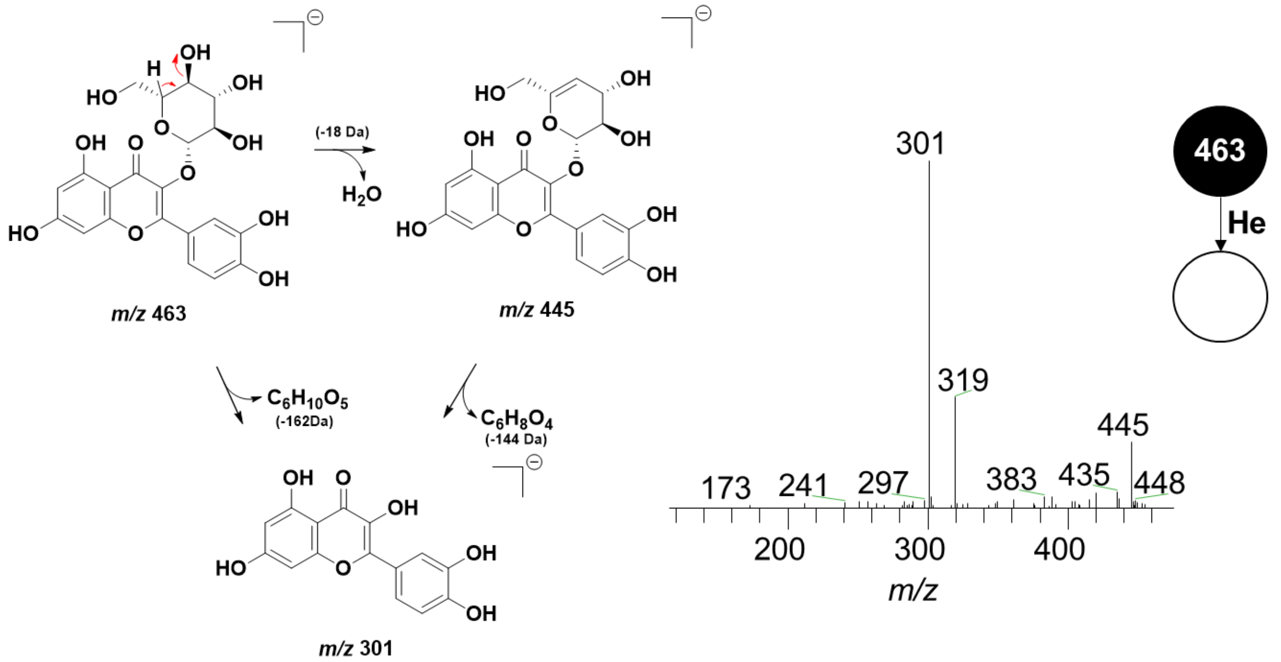

Figure S14. ESI(-)MS^2 mass spectrum and fragmentation mechanism of quercetin 3-*O*-glucoside.

Table S6. Fragments in ESI(-)MS^2 of organic acids

ID	Compound	*m/z*	MS^2
1	Quinic acid	191	127(100), 85(89), 173(88), 93(47), 111(33), 171(25), 87(20)
48	Cumaric acid	163	119(100)
49	D-gluconic acid	195	129(100), 177(28), 159(20), 99(5), 75(2)
50	Hydroxy-methoxybenzoic acid (1)	167	123(100), 153(98), 108(9), 149(6), 139(5)
51	Hydroxy-methoxybenzoic acid (2)	167	152(100), 123(74), 108(11)
52	Hydroxy-methoxybenzoic acid (3)	167	152(100), 123(96), 108(13)
53	Malic acid	133	115(100), 87(3), 71(2)
54	Salicylic acid	137	93(100)
55	Caffeoylvaleroylquinic acid	437	275(100)
